# Proteome-wide genetic analyses identify ASGR1 as a potential biomarker linking smoking with ischemic heart disease

**DOI:** 10.1038/s44161-026-00871-x

**Published:** 2026-09-22

**Authors:** Alexander C. Tinworth, Pang Yao, Andri Iona, Alfred Pozarickij, Adam Von Ende, Iona Y. Millwood, Robin G. Walters, Robert Clarke, Fiona Bragg, Zhengming Chen

**Affiliations:** 1 Clinical Trial Service Unit and Epidemiological Studies Unit (CTSU), Nuffield Department of Population Health, University of Oxford, Oxford, UK; 2 Health Data Research UK Oxford, University of Oxford, Oxford, UK

**Keywords:** Vascular diseases, Genetics research, Drug development

## Abstract

Tobacco smoking increases the risk of multiple diseases, but the biological mechanisms involved remain incompletely understood. Here we characterize smoking-related protein profiles to identify potentially causal biomarkers mediating smoking’s effects. In European-ancestry populations, genetically predicted smoking exposures were significantly associated with levels of 93 (total: 2,922) proteins, including higher levels of asialoglycoprotein receptor 1 (ASGR1). The positive association of ASGR1 levels with ischemic heart disease (IHD) was supported by colocalization (posterior probability of hypothesis 4 = 0.91), and was estimated to explain 6.35% (95% confidence interval 2.61–10.09%, *P* = 8.77 × 10^−4^) of smoking’s effects on IHD in two-step Mendelian randomization analyses. In network analyses, genetically predicted ASGR1 levels were inversely associated with plasma levels of more than 100 proteins, including growth arrest-specific 6, with all these proteins having rs55714927 (that is, a *cis*-protein quantitative trait locus for ASGR1) as a *trans*-protein quantitative trait locus. Sensitivity analyses in never-smokers identified a nonstatistically significant residual association between ASGR1 and IHD, which is consistent with possible horizontal pleiotropy, with body mass index implicated as a potential contributing pathway.

## Abstract (web summary)

Tinworth et al. identify ASGR1 as a potential molecular mediator of smoking-associated risk of ischemic heart disease.

## Main

Tobacco smoking remains a leading cause of global morbidity and mortality, accounting for over eight million deaths and a substantial attributable fraction of ischemic heart disease (IHD), stroke and chronic obstructive pulmonary disease (COPD)^1–4^. Despite recent declines in smoking rates, there are an estimated one billion tobacco smokers worldwide^5^. The associations of smoking with IHD and many other diseases are well established. However, the molecular mechanisms through which smoking drives disease development remain incompletely understood. Cigarette smoke contains more than 9,500 chemicals, many of which may induce cellular injury, alter gene expression and perturb protein synthesis, secretion or leakage into the circulation^6,7^. Clinical and experimental evidence implicates oxidative stress, endothelial dysfunction, immune-mediated vascular inflammation, thrombosis and vascular calcification in atherogenesis and IHD pathogenesis^8–10^. However, the extent to which specific circulating plasma protein biomarkers mediate, reflect or accelerate these processes remains unclear. As such, proteome-wide investigation may identify important new molecular biomarkers linking smoking exposure to downstream disease risk.

Advances in affinity-based proteomics have enabled large-scale quantification of thousands of circulating plasma proteins in population-based studies^11^. Recent analyses in the UK Biobank (UKB) have suggested that more than half of the ~3,000 Olink plasma proteins differ between smokers and nonsmokers^12^. However, the causal nature of these associations, and the extent to which smoking-related proteins interact with each other to affect and mediate disease risk, remain to be elucidated. Mendelian randomization (MR)^13–15^, which uses genetic variants to mitigate residual confounding and reverse causation inherent to observational studies, provides a framework for strengthening causal inference^16^. In this context, genetic variants associated with circulating protein levels, termed protein quantitative trait loci (pQTLs)^17,18^, can be used as instruments to proxy protein abundance and evaluate their causal effects on disease risk. This provides a powerful framework for investigating proteomic mediators of complex exposures such as smoking.

Using an integrative approach and data from the UKB and other European studies, this study aimed to elucidate the proteomic consequences of smoking and the potential mediating effects of protein biomarkers on disease etiology. We first applied proteome-wide and *cis*-MR approaches to identify proteins influenced by smoking and to assess their potential causal roles in disease pathogenesis. We then integrated Bayesian colocalization, multivariable MR (MVMR) and replication in independent cohorts to strengthen causal inference. Finally, we undertook phenome-wide association studies (PheWAS) and mediation analyses to clarify the mechanistic roles of specific identified protein biomarkers in smoking-disease associations.

## Results

### Study overview

**Fig. 1:**
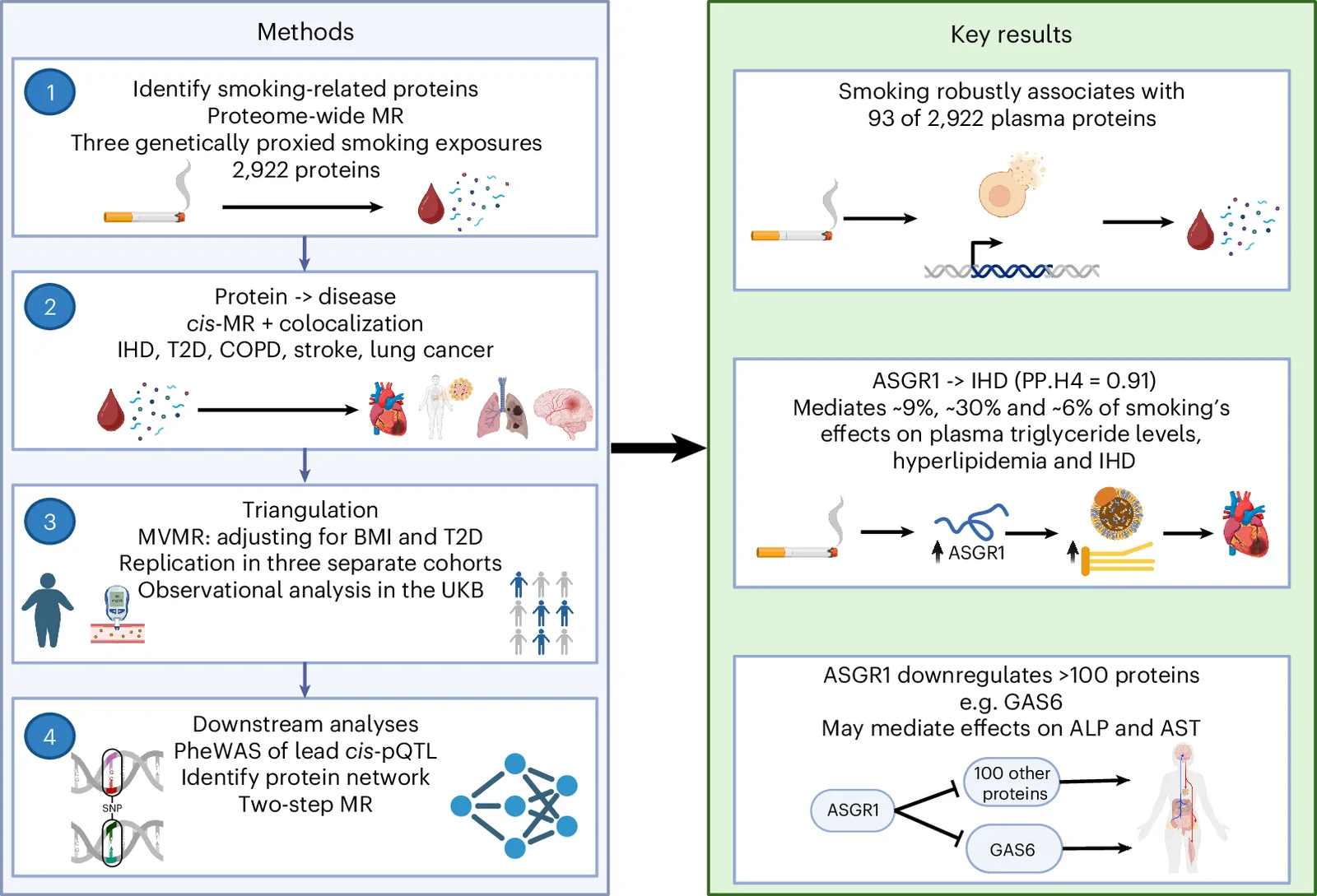
Summary of the study design and key results. Illustration created in BioRender; Tinworth, A. https://biorender.com/wyalqt7 (2026).

This study was conducted in four sequential, a-priori-defined stages, according to established analytical frameworks, and is summarized in Fig. 1 (refs. ^19,20^). In stage 1, we performed proteome-wide MR using three complementary genetic instruments for smoking initiation (GI_SmkInit_), the lifetime smoking index (GI_LSI_) and cigarettes smoked per day (GI_CigDay_), against 2,922 circulating plasma proteins. Proteins supported by sensitivity analyses, alternative smoking instruments, reverse-MR analyses and a negative control analysis in never-smokers were advanced to stage 2. In stage 2, we tested smoking-related proteins for causal associations with five smoking-related diseases (IHD, type 2 diabetes (T2D), COPD, ischemic stroke and lung cancer), using *cis*-MR and Bayesian genetic colocalization^21–25^. In stage 3, proteins showing robust evidence of disease association were further evaluated by (1) MVMR adjusting for vascular risk factors with potentially overlapping genetic architecture to smoking, (2) replication in independent proteomic cohorts, (3) integration of tissue-specific QTLs and (4) triangulation through observational analyses in the UKB. In stage 4, we characterized downstream phenotypes associated with lead *cis*-pQTLs using phenome-wide association analyses (PheWAS) and applied two-step MR to quantify protein-mediated effects and identify protein–protein networks. All contributing studies received approval from their respective institutional review boards; all participants provided informed consent. The datasets contributing to each analysis are provided in Supplementary Table 1.

### Identification of smoking-related proteins (stage 1)

To characterize the effects of smoking on the circulating proteome, we tested genetic instruments for smoking initiation (GI_SmkInit_), GI_LSI_ and GI_CigDay_ against 2,922 plasma proteins in the UKB Pharma Proteomics Project (PPP). The three instruments included 248 (mean F-statistic: 38.0, 0.4% variance explained), 126 (44.1, 1.2%) and 53 (83.4, 0.7%) single-nucleotide polymorphisms (SNPs), respectively (Supplementary Table 2).

**Fig. 2:**
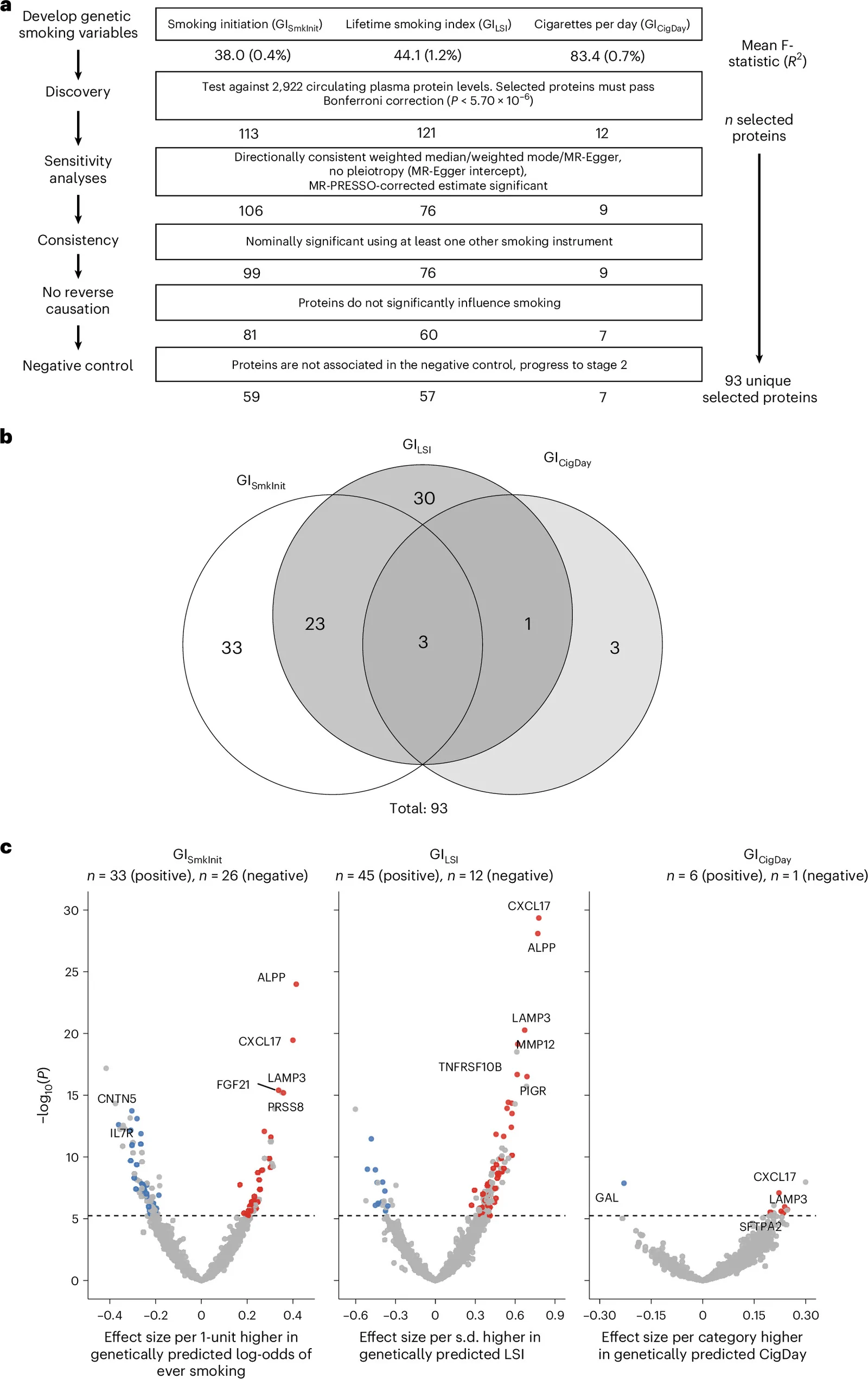
Proteome-wide MR of smoking behavior on circulating plasma proteins. **a**, Flow diagram summarizing the stage 1 analyses and sequential filtering steps used to identify smoking-related plasma proteins. **b**, Venn diagram displaying the overlap of smoking-related proteins identified using each genetic smoking instrument after all filtering criteria were applied. **c**, Volcano plots displaying MR effect estimates (random-effects inverse-variance-weighted (RE-IVW) method) for each smoking instrument on plasma protein levels. Proteins selected after all filtering steps are highlighted in red (positive associations) and blue (negative associations). Gray points represent proteins not meeting the selection criteria. Selection criteria included Bonferroni-corrected significance (*P* < 5.70 × 10^−6^), directional consistency across sensitivity analyses (weighted median, weighted mode, MR-Egger), absence of horizontal pleiotropy (MR-Egger intercept *P* ≥ 0.05), robust to outlier correction using MR-PRESSO (*P* < 0.05), nominal significance (*P* < 0.05) using at least one alternative smoking instrument, no evidence of reverse causation in bidirectional MR (*P* < 0.05/324) and nonsignificant in the negative control analyses (*P* < 0.05/108). All reported *P* values are two-sided. PRESSO, Pleiotropy RESidual Sum and Outlier.

Proteome-wide MR identified 173 unique proteins associated with at least one smoking instrument after Bonferroni correction (*P* < 5.70 × 10^−6^; Fig. 2). We then applied prespecified sensitivity and filtering criteria to prioritize proteins most likely to reflect the effects of smoking rather than pleiotropy or reverse causation. We excluded 32 proteins that were not directionally consistent in MR sensitivity analyses, seven that lacked nominal support of association with an alternative smoking instrument, 26 with evidence of association with smoking in reverse-MR and 15 that failed the negative control in never-smokers. Therefore, 93 unique proteins were selected for progression to stage 2 (Supplementary Tables 3–7). Of these, GI_SmkInit_ was associated with 59 proteins, GI_LSI_ with 57 and GI_CigDay_ with seven. Three proteins (chemokine (C-X-C motif) ligand 17, LAMP3, SFTPA2) showed consistent attenuation across all three smoking instruments.

**Extended Data Fig. 1:**
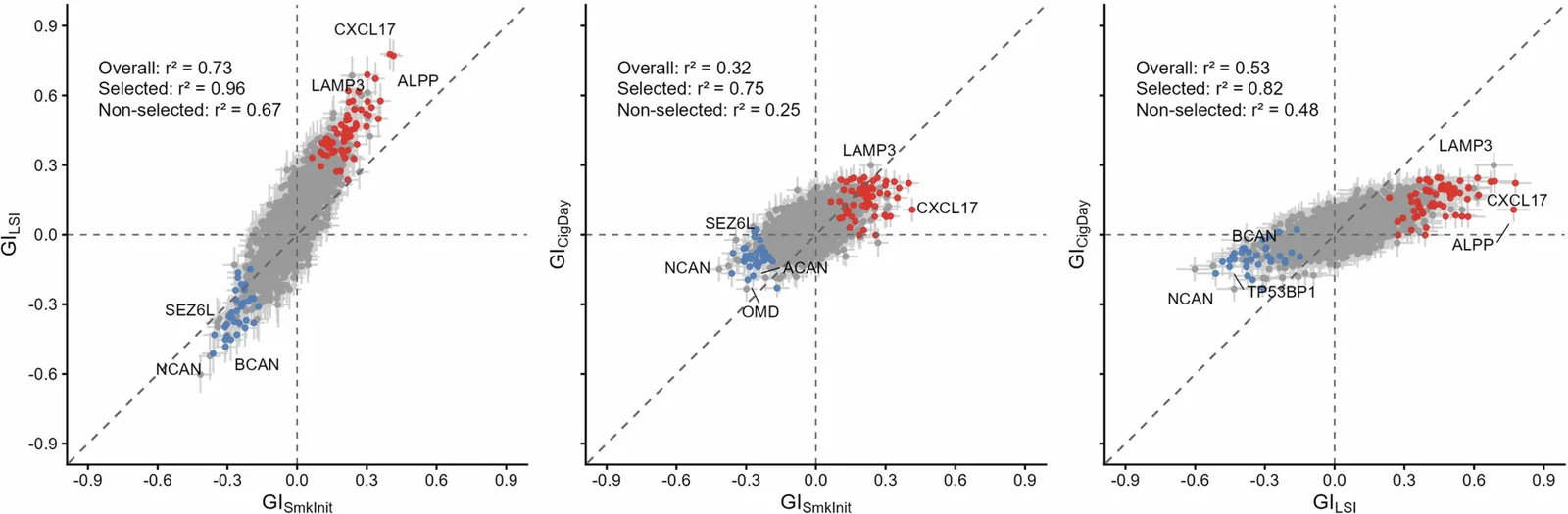
Pairwise correlations of MR estimates between different genetic instruments for smoking behaviour Comparisons are shown for GI_SmkInit_ vs GI_LSI_, GI_SmkInit_ vs GI_CigDay_, and GI_LSI_ vs GI_CigDay_. Each point represents MR estimates between genetically-proxied smoking and circulating protein levels. Red (positive associations) and blue (negative associations) points indicate proteins that passed all filtering steps and progressed to Step 2, with grey representing proteins not meeting selection criteria. Dashed diagonal lines denote equality (y = x) and horizontal/vertical lines denote beta = 0. *r*^*2*^ values correspond to Pearson correlation coefficients between effect estimates obtained using each pair of genetic instruments. Abbreviations: GI_CigDay_=genetic instrument for cigarettes per day, GI_LSI_=genetic instrument for the lifetime smoking index; GI_SmkInit_=genetic instrument for smoking initiation; MR=Mendelian randomization.

MR effect estimates of all 2,922 proteins were strongly correlated between GI_SmkInit_ and GI_LSI_ (Pearson *r*^*2*^ = 0.73), moderately correlated between GI_LSI_ and GI_CigDay_ (*r*^*2*^ = 0.53) and weakly correlated between GI_SmkInit_ and GI_CigDay_ (*r*^*2*^ = 0.32) (Extended data Fig. 1). Restriction to the 93 proteins advanced to stage 2 revealed strong concordance across all instruments (*r*^*2*^ ≥ 0.75).

**Fig. 3:**
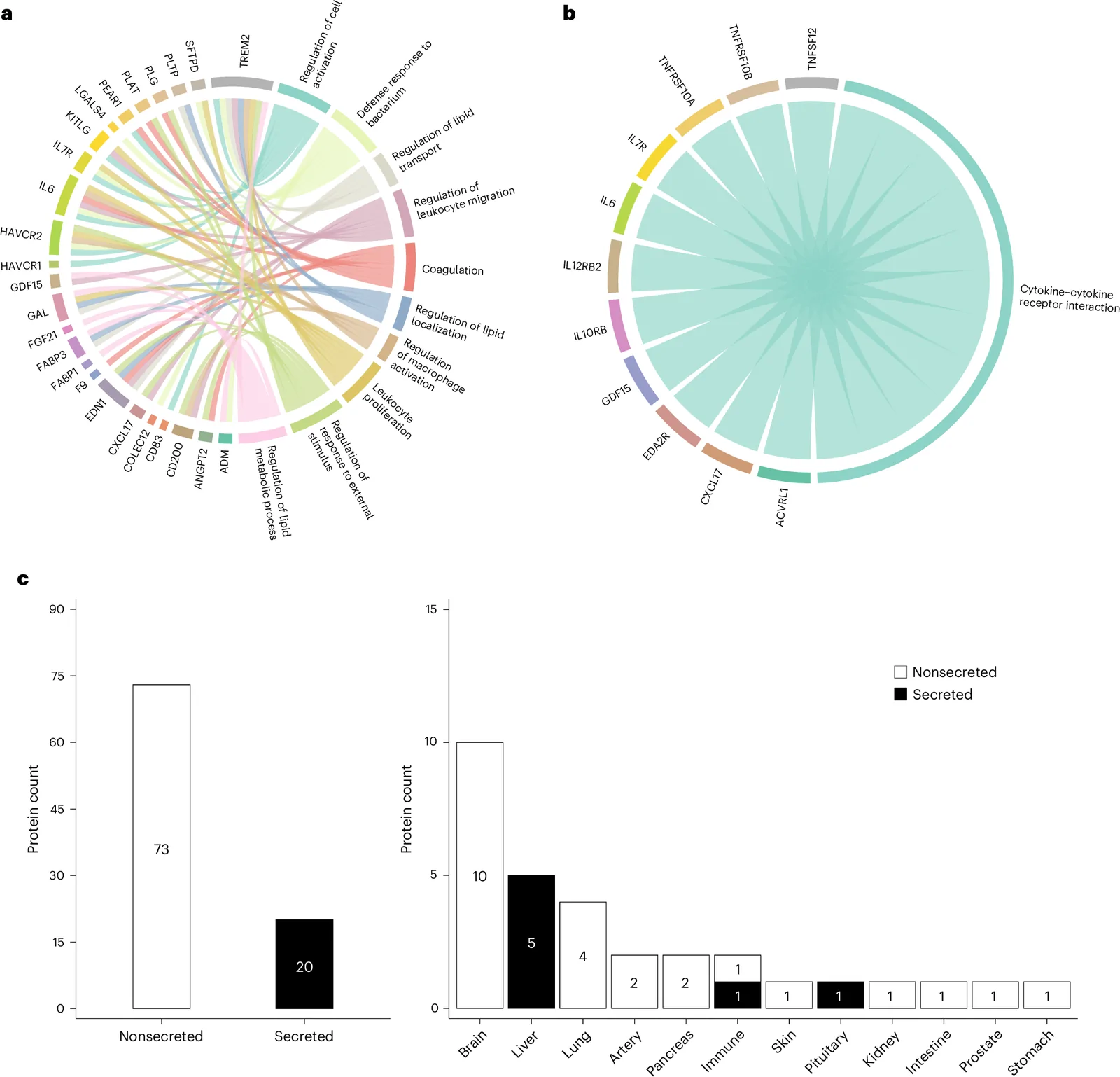
Functional enrichment analyses, secretion status and organ expression data on 93 smoking-related proteins. **a**, Chord diagram showing enriched Gene Ontology (GO) Biological Process terms for the 93 smoking-related proteins identified in stage 1. The right semicircle displays the top ten significantly enriched GO Biological Processes, while the left semicircle lists contributing proteins. Chords indicate protein–term relationships. **b**, Chord diagram illustrating enriched Kyoto Encyclopedia of Genes and Genomes (KEGG) pathways for the same protein set. The right semicircle shows one significantly enriched KEGG pathway; the left semicircle indicates proteins contributing to this pathway. Enrichment analyses in **a** and **b** were performed using hypergeometric testing with FDR correction (two-sided *P* < 0.05). **c**, Bar charts summarizing the secretion status and tissue expression patterns of smoking-related proteins. Left: Secretion status derived from the Human Protein Atlas, classifying proteins as secreted or nonsecreted. Right: Organ-enriched expression among smoking-related proteins (*n* = 31), assessed using the GTEx. Tissue enrichment was defined as fourfold or greater higher expression in one organ relative to all others.

We next examined whether the smoking-related proteins were enriched for biologically relevant pathways. Functional enrichment analyses showed overrepresentation of smoking-relevant biological processes, including regulating responses to external stimuli, macrophage activation and cytokine–cytokine receptor interactions (false discovery rate (FDR) < 0.05; Fig. 3). These signals were primarily driven by proteins attenuated by GI_SmkInit_. Restriction to proteins identified using GI_LSI_ additionally highlighted regulation of the mitogen-activated protein kinase cascade and noncanonical nuclear factor kappa-light-chain enhancer of activated B cell signal transduction (Supplementary Table 8). Human Protein Atlas annotations classified 20 proteins as actively secreted into the plasma, while Genotype-Tissue Expression (GTEx) Project data identified 31 proteins with organ-enriched expression, including ten in the brain, five in the liver and four in the lung.

### Associations of smoking-related proteins with risk of multiple diseases (stage 2)

We next assessed whether the 93 smoking-related proteins influenced five major smoking-related diseases. All three smoking instruments were associated with a higher disease risk (Supplementary Table 9). Of the 93 smoking-related proteins, 80 had at least one available *cis*-pQTL and were evaluated using *cis*-MR.

**Fig. 4:**
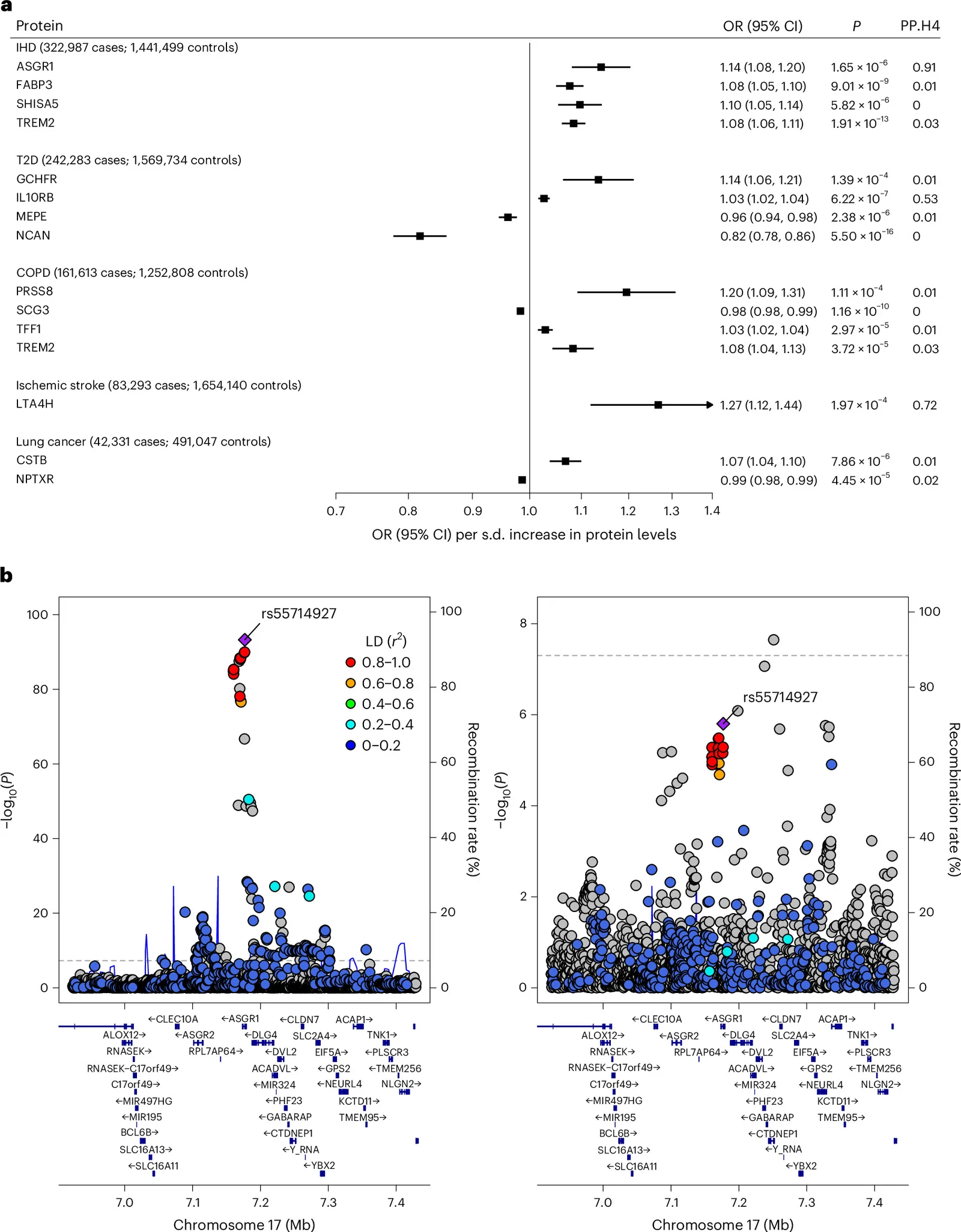
MR estimates and colocalization of smoking-related proteins with smoking-related diseases. **a**, Forest plot showing MR estimates for associations between smoking-related plasma proteins and five smoking-related disease outcomes: IHD, T2D, COPD, ischemic stroke and lung cancer. Effect estimates are presented as ORs per s.d. higher in genetically predicted circulating protein levels, with the error bars representing the 95% CIs. Only associations surpassing Bonferroni correction (*P* < 6.25 × 10^−4^) and passing the sensitivity analyses are shown. PP.H4 denotes the posterior probability of a shared causal variant from ABF colocalization; bold indicates strong evidence of colocalization (PP.H4 > 0.80). **b**, Regional association (LocusZoom) plots for the ASGR1 locus. Left: Genetic associations with circulating ASGR1 levels. Right: Associations with IHD across a ±250-kb window centered on the lead *cis*-pQTL (rs55714927). Each point represents an SNP, colored according to LD (*r*^2^) with the lead variant. All reported *P* values are two-sided.

Fifteen protein–disease associations passed Bonferroni correction (*P* < 6.25 × 10^−4^), were directionally concordant with the prior smoking–protein association and showed no evidence of horizontal pleiotropy (Fig. 4 and Supplementary Tables 10–14). Higher genetically predicted levels of ASGR1, FABP3, SHISA5 and TREM2 were associated with higher IHD risk. For T2D, higher GCHFR and IL10RB and lower MEPE and NCAN were associated with a higher risk. Higher PRSS8, TFF1, TREM2 and lower SCG3 levels were associated with COPD risk; higher LTA4H was associated with ischemic stroke; and higher CSTB and lower NPTXR were associated with lung cancer.

To distinguish shared causal signals from associations arising through linkage disequilibrium (LD), we performed Bayesian colocalization for these 15 protein–disease pairs. Approximate Bayes factor (ABF) colocalization provided strong evidence that ASGR1 (identified using GI_SmkInit_) and IHD shared a causal variant (posterior probability hypothesis 4 (PP.H4) = 0.91), whereas Sum of Single Effects (SuSiE) colocalization did not identify a shared signal for any protein–disease pair (Supplementary Table 15). Therefore, ASGR1, identified using GI_SmkInit_, was prioritized for further investigation. In two-step MR, ASGR1 was estimated to mediate 6.35% (95% CI: 2.61%, 10.09%; *P* = 8.77 × 10^−4^) of the association between GI_SmkInit_ and IHD (Supplementary Table 16).

### Vascular risk factor MVMR and replication (stage 3)

**Fig. 5:**
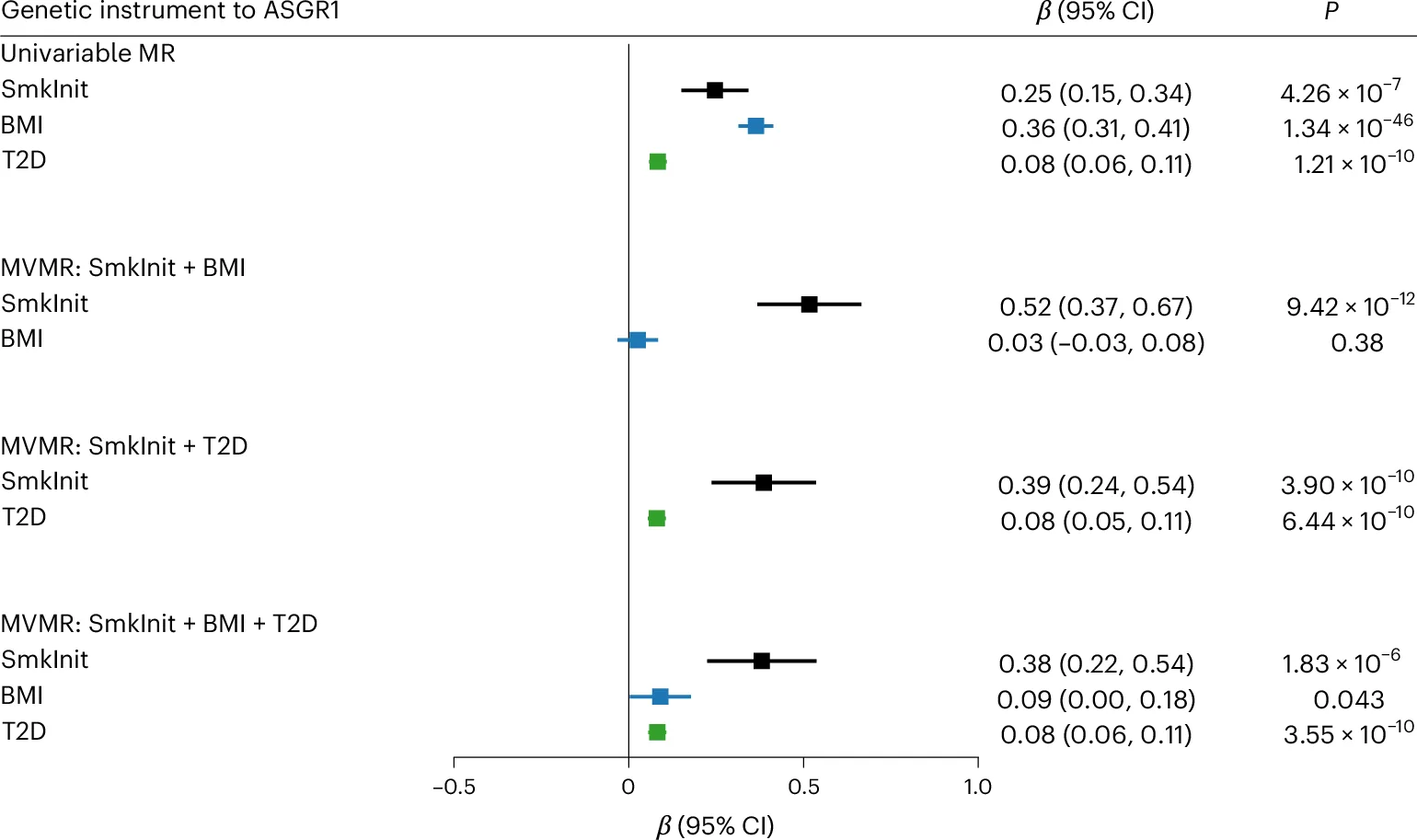
Univariable and multivariable MR estimates of smoking behavior, BMI and T2D on circulating ASGR1 levels. Forest plot showing univariable and MVMR estimates for the associations between GI_SmkInit_ (*n* = 2,669,029), BMI (*n* = 806,834), T2D (242,283 cases; 1,441,499 controls) and circulating plasma ASGR1 levels (*n* = 34,557). Effect estimates are presented as *β* coefficients per s.d. higher in BMI, per 1-unit higher genetically predicted log-odds of ever smoking (GI_SmkInit_) or per 1-unit higher genetically predicted log-odds of T2D, with the error bars representing the 95% CIs. All reported *P* values are two-sided.

Because smoking shares genetic architecture with several vascular risk factors, we evaluated potential confounding of the smoking–ASGR1 association using MVMR. Of the studied vascular risk factors (body mass index (BMI), low-density lipoprotein (LDL) cholesterol, systolic blood pressure (SBP), diastolic blood pressure (DBP), T2D), BMI (*β* = 0.36, 95% CI: 0.31, 0.41, *P* = 1.34 × 10^−46^) and T2D (0.08, 95% CI: 0.06, 0.11, *P* = 1.21 × 10^−10^) were associated with higher ASGR1 levels using univariable MR (Fig. 5). In MVMR, the association between GI_SmkInit_ and ASGR1 levels (0.25, 95% CI: 0.15, 0.34, *P* = 4.26 × 10^−7^) strengthened after adjustment for BMI (0.52, 95% CI: 0.37, 0.67, *P* = 9.42 × 10^−12^), T2D (0.39, 95% CI: 0.24, 0.54, *P* = 3.90 × 10^−10^) or both jointly (0.38, 95% CI: 0.22, 0.54, *P* = 1.83 × 10^−6^). By contrast, the BMI–ASGR1 association attenuated to the null after adjustment for smoking (0.03, 95% CI: −0.03, 0.08, *P* = 0.38) and was only nominally significant after concurrent adjustment for smoking and T2D (0.09, 95% CI: 0, 0.18, *P* = 0.043), whereas the T2D–ASGR1 association remained significant.

**Extended Data Fig. 2:**
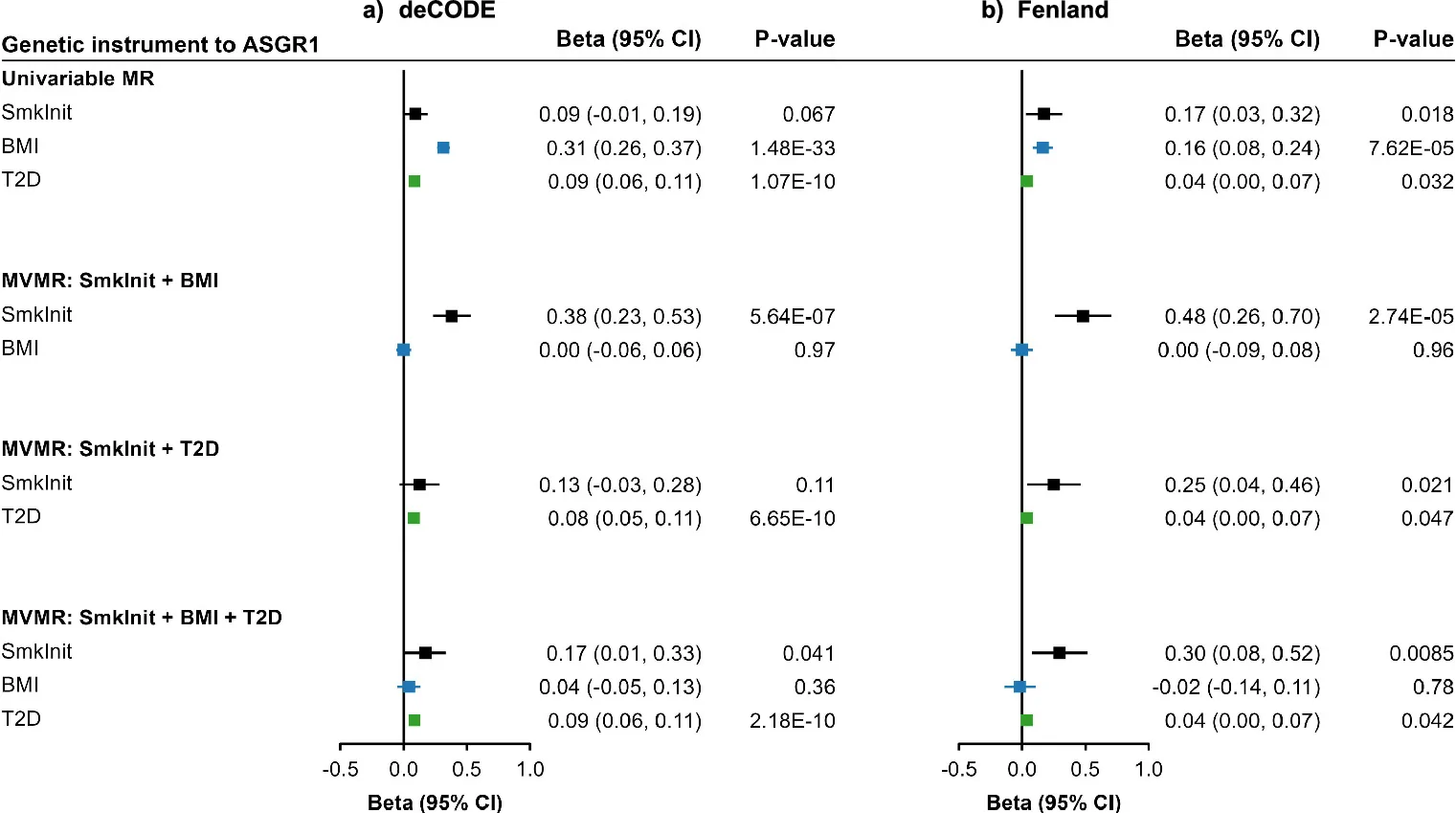
Univariable and multivariable MR estimates of smoking behaviour, BMI, and T2D on circulating ASGR1 levels, in separate cohorts. Forest plot showing univariable and MVMR estimates for the associations between GI_SmkInit_ (*n* = 2,669,029), BMI (*n* = 806,834), T2D (242,283 cases; 1,441,499 controls), and circulating plasma ASGR1 levels in (**a**) deCODE (*n* = 35,559) and (**b**) Fenland (*n* = 10,804). Effect estimates are presented as beta coefficients per standard deviation higher in BMI, per 1-unit higher genetically predicted log-odds of ever smoking (GI-SmkInit), or per 1-unit higher genetically predicted log-odds of T2D with error bars representing 95% Cis. All reported *p* values are two-sided. Abbreviations: ASGR1=asialoglycoprotein receptor 1; BMI=body mass index; CI=confidence interval; MR=Mendelian randomization; SD=standard deviation; T2D=type 2 diabetes.

We then evaluated the smoking–ASGR1 association in independent proteomic datasets. Univariable analyses provided nominal support in Fenland (*β* = 0.17, 95% CI: 0.03, 0.32, *P* = 0.018) and a directionally consistent estimate in deCODE (0.09, 95% CI: −0.01, 0.19, *P* = 0.067) (Extended Data Fig. 2 and Supplementary Table 17). Meta-analysis across UKB-PPP, Fenland and deCODE supported an overall association between smoking and higher ASGR1 levels (0.17, 95% CI: 0.11, 0.23, *P* = 4.90 × 10^−8^).

MVMR replication produced a similar pattern. Adjustment for BMI strengthened the smoking–ASGR1 association in Fenland (0.48, 95% CI: 0.26, 0.70, *P* = 2.74 × 10^−5^) and deCODE (0.38, 95% CI: 0.23, 0.53, *P* = 5.64 × 10^−7^), with associations remaining significant on additional adjustment for T2D (Fenland: 0.30, 95% CI: 0.08, 0.52, *P* = 0.0085; deCODE: 0.17, 95% CI: 0.01, 0.33, *P* = 0.041). In both cohorts, the BMI–ASGR1 association attenuated to the null after adjustment for smoking and T2D, whereas the T2D association remained significant. Conditional F-statistics for GI_SmkInit_ were greater than ten for most MVMR models, although they fell to a minimum of 9.3 when BMI was included (Supplementary Table 18).

**Extended Data Fig. 3:**
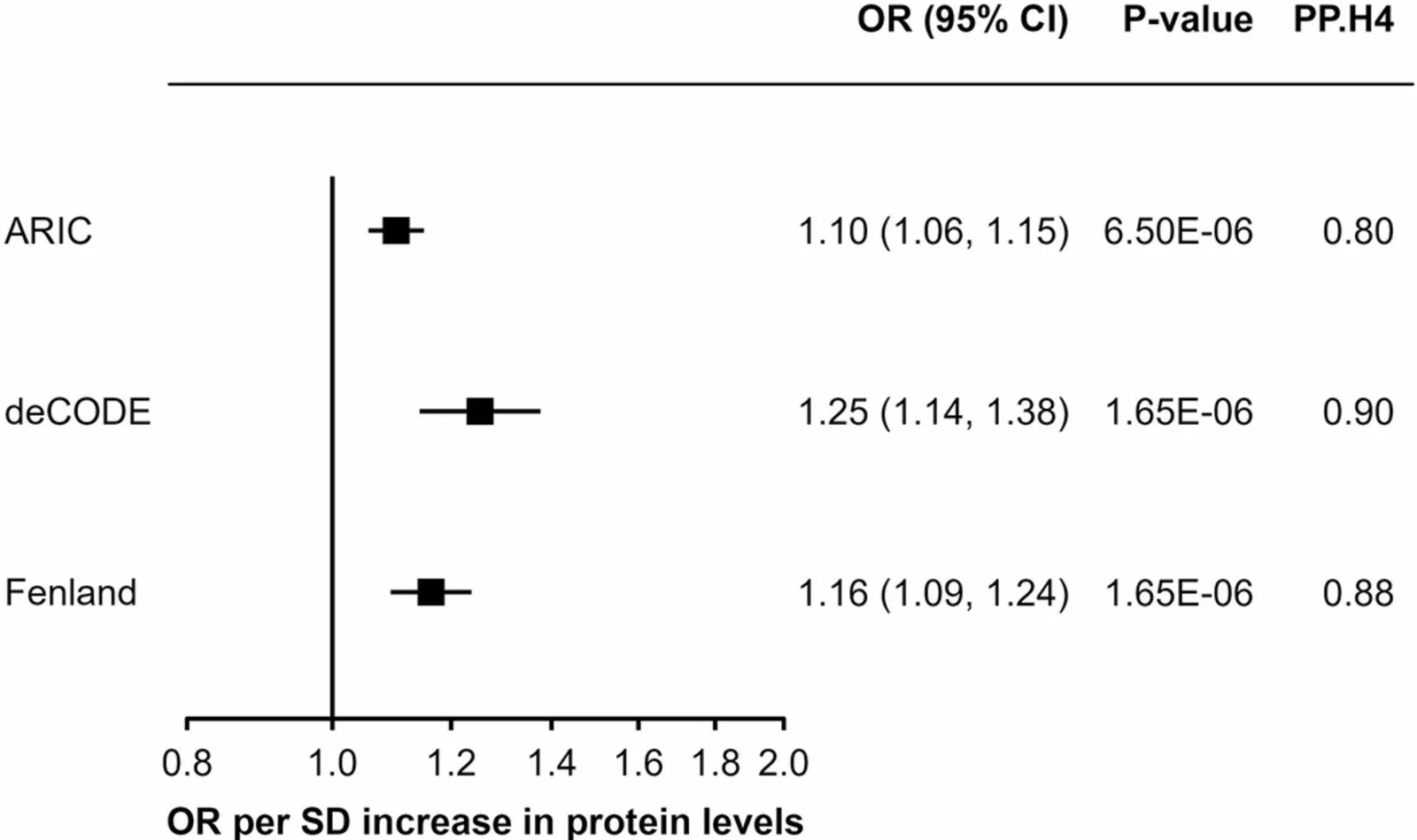
MR estimates of ASGR1 and IHD, using plasma protein levels from 3 separate cohorts. Forest plot showing MR estimates for associations between ASGR1 and IHD. Results are shown for three independent proteomic cohorts (ARIC, deCODE, and Fenland). Effect estimates are presented as ORs per standard deviation higher in genetically-proxied circulating ASGR1 levels, with error bars representing 95% CIs. PP.H4 denotes the posterior probability of a shared causal variant from ABF colocalization. All reported *p* values are two-sided. Abbreviations: ARIC=Atherosclerosis Risk in Communities; ASGR1=Asialoglycoprotein receptor 1; CI=confidence interval; IHD=ischaemic heart disease; OR=odds ratio; SD=standard deviation.

**Extended Data Fig. 4:**
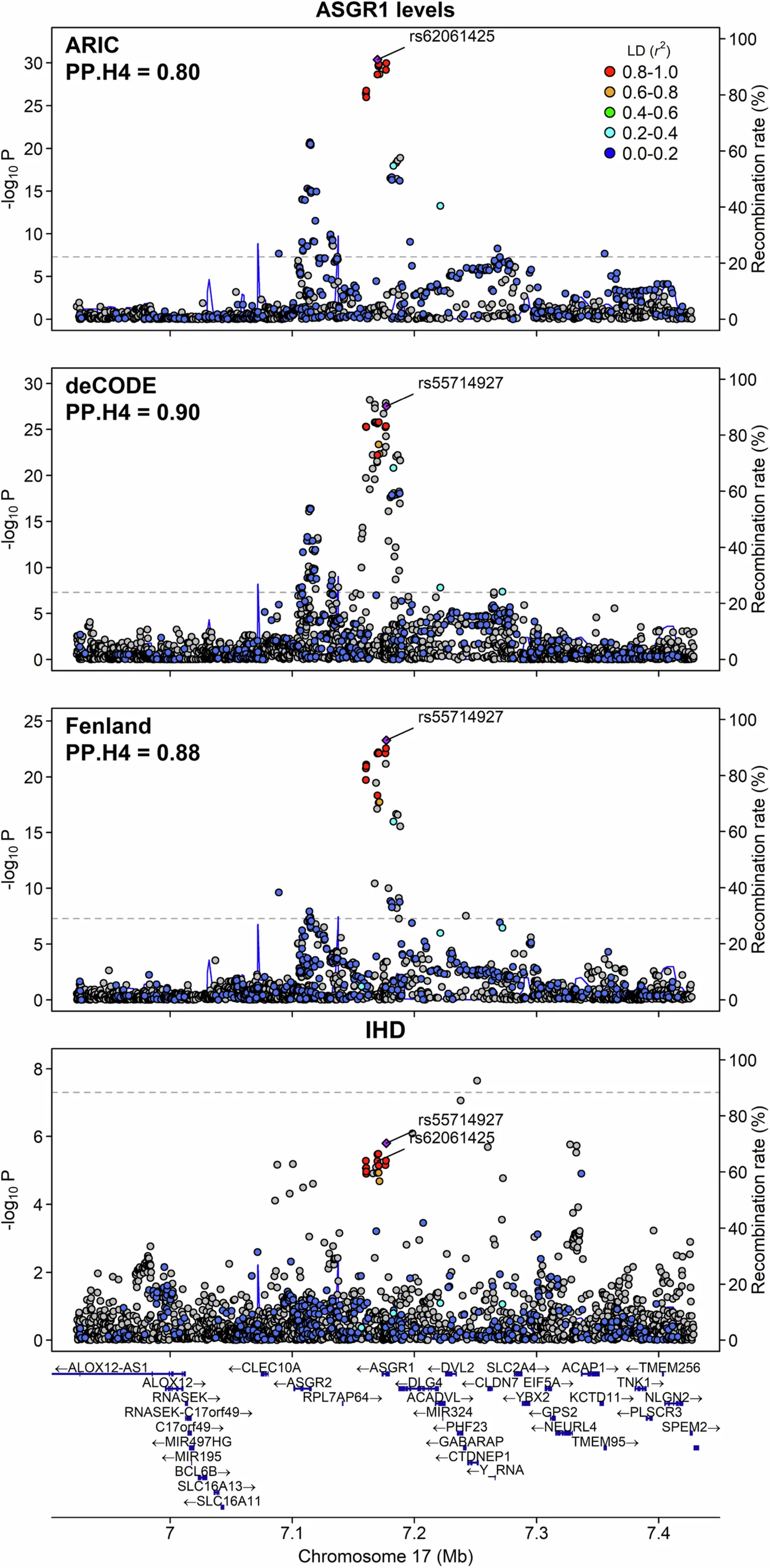
Regional association plots of ASGR1 levels from ARIC, deCODE, and Fenland, and IHD. Regional association (LocusZoom) plots for the ASGR1 locus. The associations across a ± 500 kb window were centred on the lead cis-pQTL (rs55714927 or rs62061425). Each point represents a SNP, coloured by LD (*r*^2^) with the lead variant. PP.H4 denotes the posterior probability of a shared causal variant from ABF colocalization. All reported *p* values are two-sided. Abbreviations: ARIC=Atherosclerosis Risk in Communities; ASGR1=asialoglycoprotein receptor 1; IHD=ischaemic heart disease; LD=linkage disequilibrium; P = p-value.

We also replicated the ASGR1–IHD association using independent *cis*-pQTL datasets. *cis*-pQTLs for ASGR1 from Atherosclerosis Risk in Communities (ARIC), deCODE and Fenland were each associated with higher IHD risk (*P* < 6.50 × 10^−6^), with supporting evidence from ABF colocalization (PP.H4 > 0.80) (Extended Data Figs. 3 and 4). SuSiE did not identify credible sets for IHD at the ASGR1 locus (Supplementary Table 19). Because ASGR1 showed liver-enriched expression, we additionally examined regulatory variation in GTEx. The lead ASGR1 variant, rs55714927, was a splicing QTL (sQTL) for ASGR1 at two liver splicing sites. Both splicing sites colocalized with plasma ASGR1 levels across all four proteomic studies using ABF (PP.H4 > 0.99), all except deCODE using SuSiE (PP.H4 > 0.99), and with IHD using ABF colocalization (PP.H4 = 0.91) (Supplementary Table 20).

### Observational analyses in the UKB (stage 3)

**Fig. 6:**
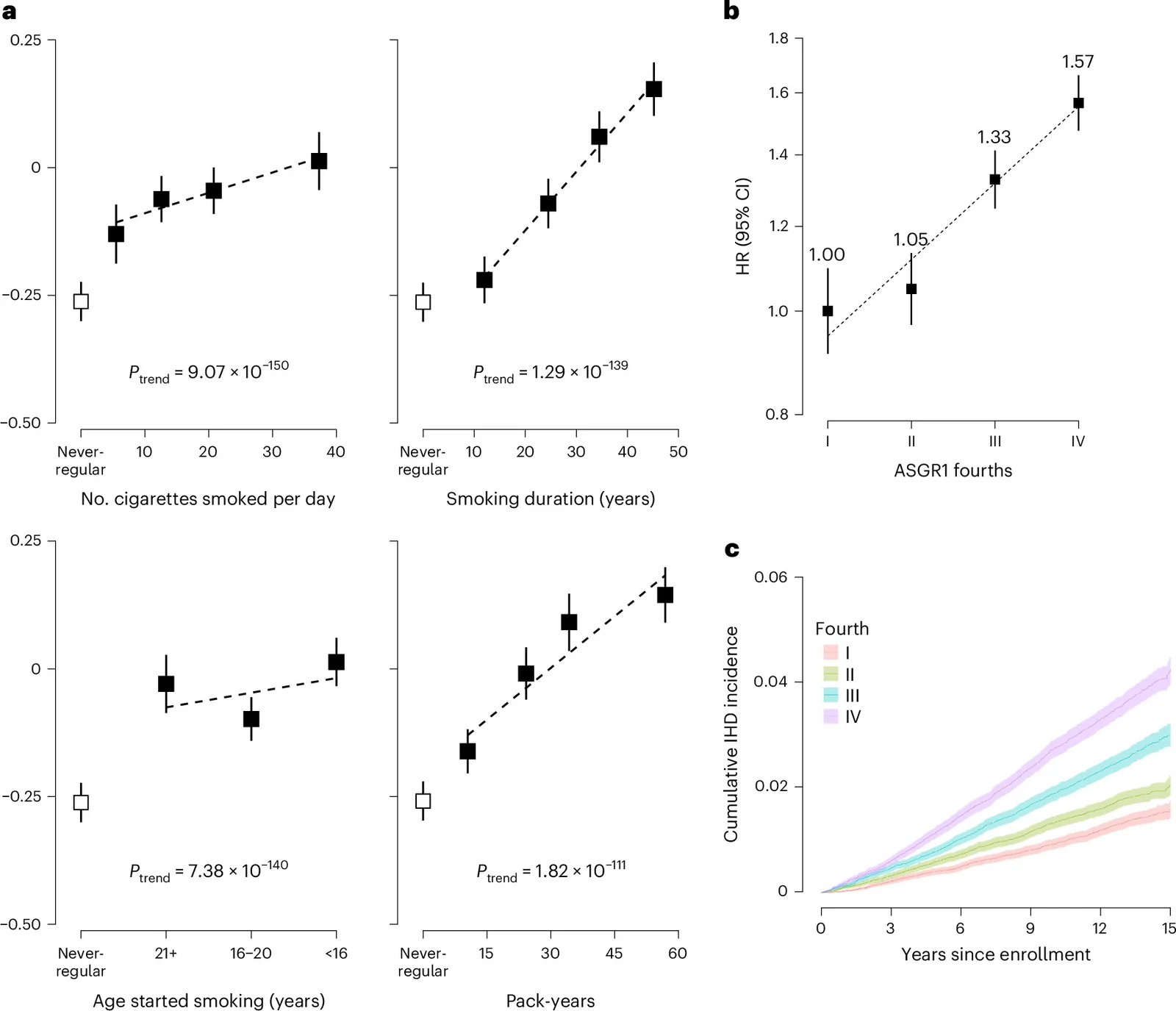
Observational associations between smoking behavior and circulating ASGR1 levels, between circulating ASGR1 levels and IHD risk, and cumulative incidence of IHD stratified according to ASGR1 levels in the UKB. **a**, Point estimates representing estimated marginal means of circulating ASGR1 levels across categories of smoking behavior. Estimates were obtained from multivariable linear regression models among 12,965 ever-regular and 24,105 never-regular smokers, adjusting for age, age^2^, sex, Townsend deprivation index, assessment center, regular alcohol consumption, BMI and fasting time. The error bars represent the 95% CIs. Tests for trend include only ever-regular smokers; reported *P* values are two-sided. **b**, Association between circulating ASGR1 fourths and incident IHD risk. Point estimates (HRs) and error bars representing the 95% CIs were estimated using Cox proportional hazards models in 40,372 participants (3,398 cases and 36,974 controls), adjusting for age at risk, sex, Townsend deprivation index, assessment center, regular alcohol consumption, BMI and smoking status. HRs are displayed on a floating absolute risk scale. **c**, Cumulative incidence of IHD stratified according to ASGR1 fourths (with higher fourths indicating higher baseline circulating ASGR1 levels). The solid center lines denote the estimated cumulative incidence; the shaded areas represent the 95% CIs. Plasma ASGR1 levels were residualized for plate effects and rank-based inverse-normal-transformed before the analysis.

We next used individual-level UKB data to triangulate genetic findings. Compared with never-regular smokers, both former (*β* = 0.13, 95% CI: 0.11, 0.15, *P* = 8.89 × 10^−31^) and current (*β* = 0.44, 95% CI: 0.41, 0.48, *P* = 3.02 × 10^−150^) smokers had higher plasma ASGR1 levels (Supplementary Table 21). Positive dose–response relationships were observed for cigarettes smoked per day (*P*_trend_ = 9.07 × 10^−150^), smoking duration (*P*_trend_ = 1.29 × 10^−139^), younger age when started to smoke (*P*_trend_ = 1.82 × 10^−111^), pack-years (*P*_trend_ = 1.50 × 10^−140^) and plasma ASGR1 levels among smokers (Fig. 6).

We then examined prospective associations between ASGR1 and incident IHD. Each standard deviation-higher ASGR1 abundance was associated with a higher risk of incident IHD (*n* = 3,398 cases; hazard ratio (HR) = 1.25, 95% CI: 1.20, 1.29, *P* = 1.68 × 10^−28^). IHD incidence was highest among participants in the highest ASGR1 quartile and lowest among those in the lowest quartile (*P*_log-rank_ = 1.45 × 10^−105^).

### Downstream analyses of ASGR1 (stage 4)

To characterize the broader phenotypic consequences of genetically predicted ASGR1 levels, we queried the lead ASGR1 *cis*-pQTL (rs55714927) in the Common Metabolic Disease Knowledge Portal (CMDKP) and Million Veteran Program (MVP). The lead variant was associated with 30 of 709 CMDKP traits after Bonferroni correction (*P* < 7.05 × 10^−5^), of which 25 met the eligibility criteria for the downstream analyses (Supplementary Table 22). No additional non-cardiometabolic phenotype reached the corresponding multiple-testing threshold in the MVP PheWAS (Supplementary Table 23). GI_SmkInit_ was associated (*P* < 0.002) with 14 of the 25 ASGR1-related traits and after prespecified filtering for pleiotropy and directional consistency with ASGR1, nine smoking–trait associations were taken forward for the mediation analyses.

Two-step MR indicated that ASGR1 mediated part of the association between GI_SmkInit_ and nine downstream phenotypes. Proportion mediated: glycated hemoglobin (HbA1c) (11.92%, 95% CI: 3.80, 20.05, *P* = 0.0040); hyperlipidemia (30.36%, 95% CI: 9.82, 50.92, *P* = 0.0038); insulin-like growth factor-1 (IGF-1) levels (14.2%, 95% CI: 5.07, 23.34, *P* = 0.0023); monocyte percentage (14.01%, 95% CI: 1.64, 26.37, *P* = 0.026); myocardial infarction (9.05%, 95% CI: 2.79, 15.30, *P* = 0.0046); nucleated red blood cell (RBC) count (20.16%, 95% CI: 6.11, 34.21, *P* = 0.0049); RBC percentage (20.00%, 95% CI: 6.13, 33.90); RBC distribution width (4.77%, 95% CI: 1.76, 7.77, *P* = 0.0019); and plasma triglycerides (8.89%, 95% CI: 3.76, 14.02, *P* = 6.80 × 10^−4^) (Table 1 and Supplementary Table 24).

**Table 1:** Genetically inferred mediation of smoking–trait associations by ASGR1

Pathway	*β* (95% CI)	*P*	Proportion mediated by ASGR1 (95% CI)	*P*
**HbA1c**				
GI_SmkInit_	0.12 (0.07, 0.16)	1.70 × 10^−7^	0.12 (0.04, 0.20)	0.004
ASGR1	0.06 (0.03, 0.08)	2.53 × 10^−6^		
**Hyperlipidemia**				
GI_SmkInit_	0.23 (0.12, 0.33)	5.69 × 10^−^^5^	0.30 (0.10, 0.51)	0.004
ASGR1	0.28 (0.20, 0.35)	1.58 × 10^−13^		
**IGF-1 levels**				
GI_SmkInit_	−0.15 (−0.21, −0.09)	5.02 × 10^−6^	0.14 (0.05, 0.23)	0.002
ASGR1	−0.09 (−0.11, −0.06)	3.32 × 10^−12^		
**Monocyte percentage**				
GI_SmkInit_	−0.08 (−0.13, −0.03)	0.0011	0.14 (0.02, 0.26)	0.026
ASGR1	−0.05 (−0.07, −0.02)	1.6 × 10^−4^		
**Myocardial infarction**				
GI_SmkInit_	0.53 (0.40, 0.66)	1.98 × 10^−15^	0.09 (0.03, 0.15)	0.005
ASGR1	0.19 (0.09, 0.29)	1.49 × 10^−4^		
**Nucleated RBC count**				
GI_SmkInit_	0.05 (0.03, 0.07)	1.13 × 10^−6^	0.20 (0.06, 0.34)	0.005
ASGR1	0.04 (0.02, 0.06)	2.56 × 10^−6^		
**Nucleated RBC percentage**				
GI_SmkInit_	0.05 (0.03, 0.07)	8.08 × 10^−7^	0.20 (0.06, 0.34)	0.005
ASGR1	0.04 (0.02, 0.06)	8.94 × 10^−9^		
**RBC distribution width**				
GI_SmkInit_	0.38 (0.32, 0.45)	7.84 × 10^−30^	0.05 (0.02, 0.08)	0.002
ASGR1	0.07 (0.04, 0.11)	2.56 × 10^−5^		
**Triglyceride levels**				
GI_SmkInit_	0.15 (0.11, 0.18)	1.56 × 10^−15^	0.09 (0.04, 0.14)	6.83 × 10^−4^
ASGR1	0.05 (0.03, 0.07)	2.11 × 10^−8^		

Traits were selected for mediation analyses if the smoking–outcome association surpassed Bonferroni correction (*P* < 0.002) and was directionally consistent with the ASGR1–outcome association. Mediation estimates were derived using a two-step MR framework, calculated as (*β*_1_ × *β*_2_) / *β*_TE_, where *β*_1_ represents the MR estimate for the smoking–ASGR1 association, *β*_2_ represents the MR estimate for the ASGR1–trait association and *β*_TE_ represents the MR estimate for the total smoking–trait association. The 95% CIs were calculated using the delta method.

Finally, we investigated whether the phenotypic effects of ASGR1 might be propagated through a broader circulating protein network. The ASGR1 *cis*-pQTL (rs55714927) was also a *trans*-pQTL for 101 circulating plasma proteins in deCODE, and genetically predicted ASGR1 levels were associated with lower levels of all 101 proteins (Supplementary Table 25). All *trans* associations were directionally consistent according to Steiger filtering, and 99 showed evidence of a shared genetic architecture (PP.H4 > 0.80) using SuSiE colocalization, although none did so using ABF colocalization. Of these, 89 network proteins had at least one available *cis*-pQTL.

Across 2,225 network protein–trait associations, 123 surpassed Bonferroni correction (*P* < 2.25 × 10^−5^). After excluding associations that were directionally inconsistent with the ASGR1–protein or ASGR1–trait associations (*n* = 55), showed evidence of reverse causation (*n* = 3) or represented inconsistent mediation (*n* = 43), 22 protein–trait associations involving 17 network proteins and nine traits were eligible for mediation analysis (Supplementary Tables 26 and 27).

**Fig. 7:**
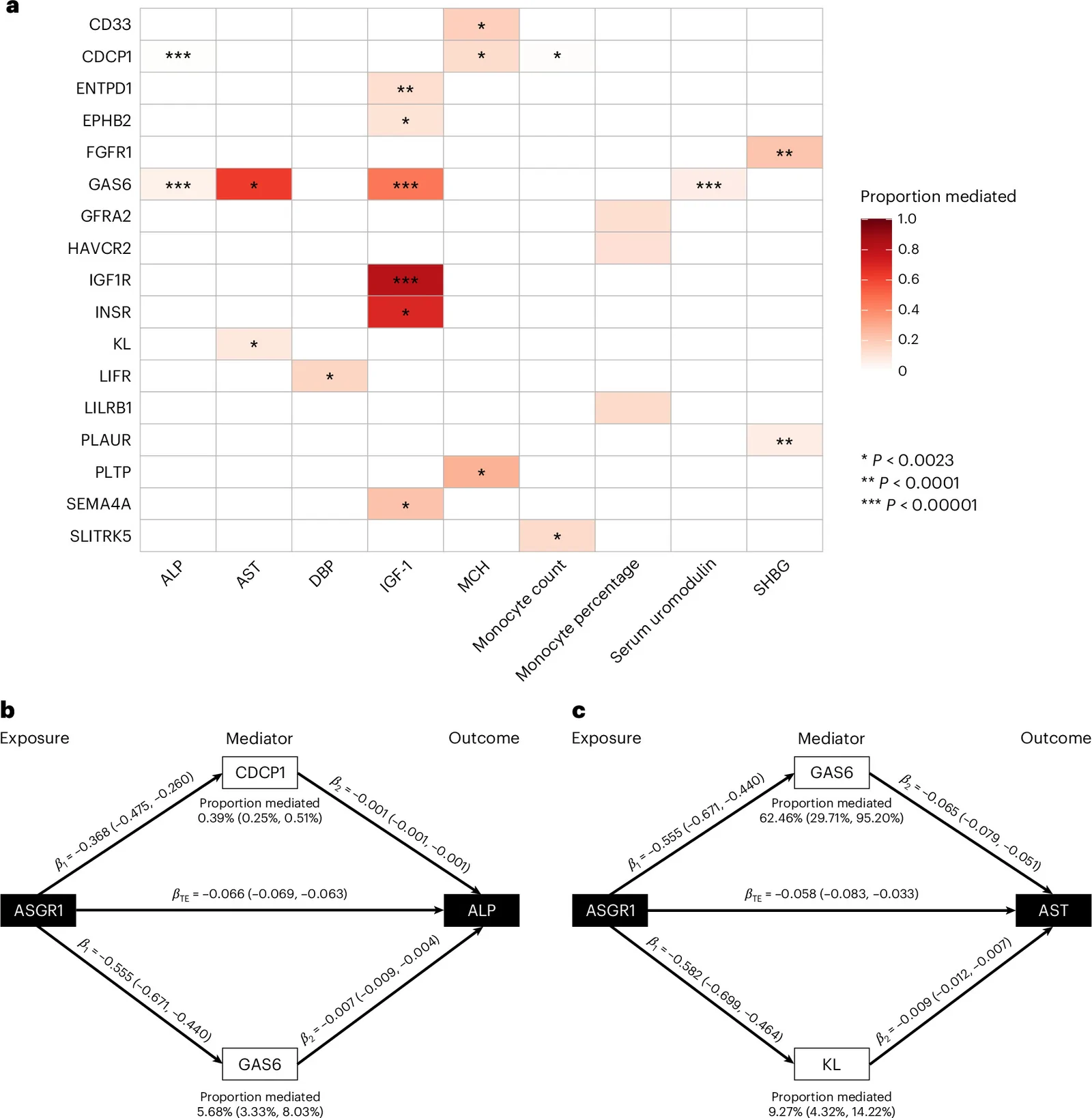
Genetically inferred mediation of ASGR1 associations with downstream traits through network proteins. **a**, Heatmap showing the estimated proportion of the association between ASGR1 and downstream traits that is mediated by individual ASGR1 network proteins. Mediation estimates were derived using a two-step MR framework, calculated as (*β*_1_ × *β*_2_) / *β*_TE_, where *β*_1_ represents the MR estimate for the ASGR1–mediator (network protein) association, *β*_2_ represents the MR estimate for the mediator–trait association and *β*_TE_ represents the MR estimate for the total ASGR1–trait association. The 95% CIs were calculated using the delta method. Color intensity reflects the proportion mediated, with darker shading indicating a larger proportion mediated. Statistical significance is denoted by asterisks (*P < 0.0023 (Bonferroni correction); **P < 0.0001; ***P < 0.00001). All reported *P* values are two-sided. **b**, Schematic illustration of the mediation of the ASGR1–ALP association by CDCP1 and GAS6, showing MR effect estimates (*β*) and corresponding 95% CIs for each step, as well as the estimated proportion mediated. **c**, Schematic illustration if the ASGR1–AST association by GAS6 and KL, presented analogously to **b**. MCH, mean corpuscular hemoglobin; SHBG, sex-hormone-binding globulin.

Of these, 19 of the 22 ASGR1–trait associations showed evidence of partial mediation by at least one network protein (Fig. 7). CUB domain-containing protein 1 (CDCP1) mediated 0.39% (95% CI: 0.26%, 0.51%; *P* = 2.13 × 10^−9^) of ASGR1’s association with alkaline phosphatase (ALP), while klotho (KL) mediated 9.27% (95% CI: 4.32%, 14.22%; *P* = 2.42 × 10^−4^) of the association between ASGR1 and aspartate aminotransferase (AST). Growth arrest-specific 6 (GAS6)-mediated ASGR1’s association with ALP (5.7%, 95% CI: 3.33%, 8.03%, *P* = 2.09 × 10^−6^) and AST (62.5%, 95% CI: 29.71%, 95.20%, *P* = 1.85×10^−4^), in addition to serum IGF-1 and uromodulin levels (Supplementary Table 28).

## Discussion

In these comprehensive analyses of the effect of tobacco smoking on the plasma proteome, we identified 93 plasma proteins robustly influenced by smoking behavior. Of these, 15 were associated with smoking-related diseases, but only the association between ASGR1 and IHD was supported by colocalization, indicating robustness to confounding by LD and hence a potentially causal association. MVMR adjusting for BMI alone and concurrently with T2D strengthened the smoking–ASGR1 association, an effect that was replicated, in addition to ASGR1’s association with IHD, across independent proteomic cohorts. In addition, ASGR1 appeared to partially mediate smoking’s effects on several cardiometabolic traits, including hyperlipidemia and triglyceride levels. Together, these findings implicate ASGR1 as a plausible molecular intermediary linking smoking exposure to downstream cardiovascular risk and traits. Furthermore, ASGR1 shared genetic architecture with a broad network of circulating proteins, several of which may also contribute to mediating the downstream effects of ASGR1.

The identification of 93 smoking-related proteins highlights the extensive systemic impact of tobacco exposure on the circulating proteome. Among these, 31 showed organ-enriched expression and 20 were actively secreted into the plasma, reflecting both tissue injury and smoking-induced alterations in gene transcript splicing and expression, potentially mediated by epigenetic mechanisms. This set included proteins previously reported to be influenced by smoking, such as growth/differentiation factor 15 (ref. ^26^), interleukin-6 (ref. ^27^), chemokine (C-X-C motif) ligand 17 (ref. ^28^) and pro-adrenomedullin^29^, as well as proteins not previously implicated, including ectodysplasin A2 receptor, vanin 1 and osteomodulin. Notably, ten organ-enriched proteins showed predominant expression in the brain, supporting accumulating evidence that smoking exerts direct effects on brain structure and function^30^. Functional enrichment analyses further linked smoking-related proteins to pathways involved in lipid transport, macrophage and leukocyte migration, regulating responses to external stimuli (defined as a change in state or activity of a cell because of an external stimulus) and cytokine–cytokine receptor interactions, processes potentially reflecting or contributing to oxidative stress, endothelial damage and immune-related inflammatory pathways that underlie smoking-related disease pathogenesis^31–33^.

Despite identification of a large number of proteins related to smoking, only ASGR1’s positive association with IHD was supported by colocalization and was estimated to mediate 6.4% of smoking’s effect on IHD. A previous observational clinical study in 32 individuals reported that smokers with metabolic dysfunction-associated steatotic liver disease had higher ASGR1 levels in circulating extracellular vesicles than nonsmoking patients with metabolic-dysfunction-associated steatotic liver disease, supporting a potential role for smoking in altering ASGR1 levels^34^. Smoking has been reported to induce widespread epigenetic and transcriptional changes, including alternative gene splicing in liver-related genes where ASGR1 is predominantly expressed, which may result in higher circulating ASGR1 levels^35,36^. This was further supported by the ASGR1 *cis*-pQTL colocalizing with two splicing sites for ASGR1 in liver tissue, potentially influencing isoform abundance or gene transcript stability and thereby altering circulating ASGR1 levels.

Moreover, ASGR1 has been repeatedly implicated in cardiovascular risk in large-scale genetic and epidemiological studies^37–39^, including loss-of-function variants associated with substantial reductions in IHD^40^. Mechanistically, ASGR1 regulates lipid metabolism via modulation of mechanistic target of rapamycin complex 1 and AMP-activated protein kinase signaling, promoting lipogenesis and impairing cholesterol efflux through increased lysosomal degradation of asialoglycoproteins^41^, effects independent of proprotein convertase subtilisin/kexin type 9 (ref. ^42^). Although SuSiE colocalization did not identify a credible set for IHD at the ASGR1 locus, the convergence of evidence from ABF colocalization, MR across multiple cohorts, observational analyses and prior literature supports a causal role for ASGR1 in IHD pathogenesis. Importantly, the ASGR1 *cis*-pQTL (rs55714927) colocalizing with two splicing sites in the liver for ASGR1 reduces the likelihood that epitope-binding artifacts explain the observed associations.

Although the association of GI_SmkInit_ with ASGR1 passed negative control filtering, some evidence of horizontal pleiotropy remained. Of the studied vascular risk factors, BMI and T2D were associated with higher ASGR1 levels, which is consistent with previous work implicating ASGR1 as a ‘BMI-driven’ protein^19^. Therefore, BMI-related pathways may have explained this residual association in never-regular smokers, given that smoking and BMI are correlated traits with partially overlapping genetic architecture. Indeed, adjustment for BMI in MVMR analyses strengthened the smoking–ASGR1 association while attenuating the BMI–ASGR1 association toward the null. Although the BMI association with ASGR1 levels in the UKB remained nominally significant after mutual adjustment for BMI and T2D, this was not replicated in deCODE or Fenland. Similarly, while the univariable smoking–ASGR1 association did not reach statistical significance in deCODE, effect estimates were directionally consistent across cohorts, significant when meta-analyzing with UKB-PPP and Fenland, and strengthened after BMI adjustment, mirroring findings in the UKB and Fenland. Together, these findings suggest that genetic instruments for smoking and BMI may capture overlapping or correlated pathways that partially obscure smoking–ASGR1 associations in univariable analyses. Although ASGR1 is probably influenced by determinants beyond smoking alone (for example, T2D), our findings support smoking exposure as a potential contributor to ASGR1 regulation and suggest that ASGR1 may represent one pathway linking smoking to IHD risk. More broadly, these findings highlight the importance of accounting for correlated vascular risk factors in proteome-wide MR studies and demonstrate how multivariable modeling can refine the interpretation of smoking-associated proteomic signatures.

The PheWAS using the CMDKP and MVP indicated that ASGR1 is genetically associated with multiple cardiometabolic traits, many of which are influenced by smoking. Our mediation analyses suggested that ASGR1 partially mediates smoking’s effects on triglyceride levels and hyperlipidemia, traits previously reported to be affected by both smoking and ASGR1, which provide a plausible pathway through which ASGR1 contributes to smoking-related IHD^43–45^. ASGR1 also appeared to mediate smoking-related variation in HbA1c; however, its role in glycemic disease remains uncertain^46^. In previous reports, ASGR1 was a weak predictor of T2D; no association with T2D was observed in the present study, despite smoking’s established contribution to T2D risk^47,48^. This ambiguity is further compounded by ASGR1’s associations with erythrocyte traits, including RBC count and distribution width, which may influence HbA1c independently of glucose homeostasis^49,50^. In contrast, our analyses revealed that genetic liability to T2D was robustly associated with circulating ASGR1 levels, even when adjusting for BMI and smoking, which may be the result of similar or overlapping mechanisms to that of smoking itself. Together, these findings suggest that ASGR1’s metabolic effects may be more closely linked to lipid metabolism and erythrocyte biology than to glycemic regulation; the precise mechanisms by which T2D raises ASGR1 levels, and how this compares to smoking-related mechanisms, require further research.

As reported previously^51^, one of the more striking observations in our analyses was the extensive downregulatory network associated with ASGR1, whereby genetically predicted higher ASGR1 levels were linked to lower circulating levels of many proteins. This network included Golgi protein 73, a protein previously reported to increase after ASGR1 inhibition, providing independent biological support for the observed network structure^52^. Several network proteins emerged as potential mediators of ASGR1’s downstream effects, including GAS6 in relation to ALP, AST, IGF-1 and uromodulin, and CDCP1 in relation to ALP, mean corpuscular hemoglobin and monocyte count. GAS6 has also been proposed as a therapeutic target in liver disease^53–55^. While these findings probably represent only a subset of ASGR1’s broader regulatory influence, they highlight ASGR1 as a central node in a complex proteomic network. Notably, although increasing Golgi protein 73 after ASGR1 inhibition has been linked to endoplasmic reticulum stress and liver injury, such associations were not evident in our analyses, underscoring the context-specific nature of ASGR1-mediated effects^52^. Collectively, these results emphasize the need to consider ASGR1’s broader network effects when evaluating its potential as a therapeutic target.

The effects of ASGR1 on the liver-related traits identified in our analyses (for example, ALP and AST) warrant additional investigation. Experimental studies suggest that ASGR1 inhibition can elevate liver enzymes and induce hepatic injury^56–58^, although human evidence is mixed. A previous study reported an inverse correlation between hepatic *ASGR1* mRNA expression and plasma levels of ALP, AST, ALT and gamma-glutamyl transferase levels in patients with liver cirrhosis, which is consistent with our PheWAS findings^52^. However, ASGR1 has not been established as a causal determinant of liver disease. The *cis*-pQTL for ASGR1 showed no nominal association with nonalcoholic liver disease, cirrhosis or other hepatic conditions in the CMDKP, nor were there reported associations in previous genetic studies^38,40^. Moreover, modest elevations in liver enzymes are a recognized feature of statin therapy, yet statin-induced liver injury is rare, potentially providing a conceptual parallel for ASGR1-targeted interventions^59–61^. Encouragingly, ASGR1 has been suggested as an indication for IHD^62^; early-phase clinical studies targeting ASGR1, including the monoclonal antibody AMG-529 and the liver-directed SZN-043, have demonstrated acceptable safety profiles, although clinical efficacy for alcohol-associated hepatitis was limited in the latter and further development has been sparse^63^. ASGR1 has additionally been exploited as a delivery mechanism for RNA-based therapeutics, but whether the cardiovascular benefits of ASGR1 modulation outweigh potential hepatic risks or could be optimized through targeting downstream network proteins such as GAS6, remains an important question for future research^64^.

This study has several strengths. The use of multiple, complementary genetic smoking instruments capturing distinct dimensions of smoking behavior enabled a comprehensive assessment of smoking’s effect on the circulating plasma proteome. We further strengthened causal inference through an integrative framework combining univariable and multivariable MR, colocalization, replication in independent cohorts and triangulation with observational data. Finally, the use of large-scale, meta-analyzed outcome datasets maximized statistical power to detect associations with smoking-related diseases.

However, our study also has several limitations. First, despite efforts to maximize power, only one protein–disease association demonstrated convincing evidence of colocalization, suggesting that the other smoking-related proteomic associations with disease may be confounded by LD. Second, ASGR1 should not be interpreted as a smoking-specific biomarker, but rather a pleiotropic cardiovascular mediator that may lie downstream of smoking and linked to multiple biological pathways. Although there was little evidence of a bidirectional effect of ASGR1 levels on IHD, we cannot exclude more complex pathways. Accordingly, our findings should be interpreted as evidence that smoking influences IHD risk in part through ASGR1-related atherogenic pathways, rather than proof of a uniquely smoking-dependent mechanism. Third, conditional F-statistics for GI_SmkInit_ declined below conventional thresholds when adjusting for BMI, raising the possibility of weak instrument bias. However, the attenuation of the BMI–ASGR1 association to the null and consistent smoking–ASGR1 associations across multiple cohorts and proteomic platforms support the validity of the primary findings. Fourth, univariable analyses did not replicate the smoking–ASGR1 association in deCODE, the largest external cohort, although meta-analysis of all three cohorts supported an association between smoking and ASGR1. Additionally, although the association in deCODE strengthened after adjustment for BMI in the MVMR analyses, the conditional F-statistic for smoking dropped to 9.3; thus, the findings should be interpreted cautiously and require further validation in independent proteomic datasets. Fifth, although Steiger filtering and bidirectional MR were used to assess causal direction, the directionality between ASGR1 and its network proteins cannot be established with certainty, as illustrated by the IGF-1 receptor, where genetic evidence supported an association between the IGF-1 receptor and IGF-1 levels but not vice versa. Sixth, interpretation of mediation estimates also requires caution as MR reflects lifelong genetically predicted exposure rather than acute or time-specific perturbations^65^. Seventh, mediation analyses were restricted to directionally consistent pathways, limiting evaluation to negatively modulated ASGR1-related traits and potentially underestimating the broader network through which ASGR1 may influence lipid metabolism, cholesterol levels and IHD. Finally, although sample overlap was minimal (1.3% for SmkInit, 7.5% for the LSI and 5.6% for CigDay) in the primary analyses, some attenuation of effect estimates cannot be excluded, although analyses using smoking instruments that exclude overlapping samples (UKB and 23AndMe) produced broadly consistent findings.

In conclusion, this study identified 93 circulating plasma proteins influenced by smoking and provides convergent genetic and observational evidence implicating ASGR1 as a potential biomarker linking smoking with IHD risk. Our findings suggest that ASGR1 contributes to smoking-induced dyslipidemia and higher triglyceride levels, thereby promoting atherosclerosis and downstream myocardial infarction risk. The genetically defined ASGR1 protein network, particularly the role of GAS6, represents a promising avenue for future research to evaluate the cardiovascular benefits and potential hepatic risks of therapeutic ASGR1 modulation.

## Methods

### Ethics

The genome-wide association studies (GWAS) that contributed the summary statistics used in these analyses all received approval from their respective institutional review boards; all participants provided informed consent. Access to individual participant data from the UKB was granted after application (no. 50474; principal investigator R.G.W.). The UKB obtained ethical approval from the North West Multi-centre Research Committee (11/NW/0382); all participants provided written informed consent.

### Stage 1: effects of smoking behaviors on the circulating plasma proteome

#### Genetic instruments and proteomic data

All genetic association data were obtained from publicly available GWAS of individuals of European ancestry. We used three complementary smoking phenotypes: smoking initiation (GI_SmkInit_: *n* = 2,669,029) and cigarettes smoked per day (GI_CigDay_: *n* = 618,489) from the GWAS and Sequencing Consortium of Alcohol and Nicotine use (GSCAN)^66^, and the lifetime smoking index (GI_LSI_: *n* = 462,690) from a UKB GWAS^67^. The lifetime smoking index is a composite measure integrating smoking intensity, duration, cessation and time since quitting^68^.

Genetic associations with 2,922 circulating plasma proteins were obtained from the UKB-PPP, in which 34,557 participants were profiled using the Olink proximity extension assay platform^18^. GLIPR1 was excluded because it failed quality control.

#### Instrument selection and MR analyses

Genome-wide genetic instruments for each smoking phenotype were constructed from genome-wide-significant (*P* < 5 × 10^−8^), independent SNPs with a minor allele frequency (MAF) greater than 0.01. Independence was ensured using LD clumping (*r*^2^ < 0.001, 10,000-kb window) in PLINK, using the phase 3 European-ancestry reference panel from the 1000 Genomes Project^69,70^.

MR estimates rely on three core assumptions. Genetic instruments are: (1) robustly associated with the exposure; (2) independent of confounders of the exposure–outcome association; and (3) influence the outcome exclusively through the exposure^13^. We estimated the effects of smoking behaviors on circulating plasma proteins using the RE-IVW method. Smoking-associated SNPs within the *cis*-region of the gene encoding the protein under investigation were excluded to minimize local genetic confounding. Statistical significance was defined using Bonferroni correction for multiple testing (that is, three smoking instruments × 2,922 proteins; *P* < 5.70 × 10^−6^).

Proteins meeting this threshold were advanced to stage 2 if they demonstrated (1) directional consistency across MR sensitivity analyses (weighted median, weighted mode, MR-Egger); (2) no evidence of directional horizontal pleiotropy by the MR-Egger intercept (*P* ≥ 0.05); (3) nominal evidence of association (*P* < 0.05) using at least one alternative smoking instrument; (4) robustness to outlier exclusion using the MR-PRESSO framework (outlier-corrected *P* < 0.05); and (5) no significant association with the smoking phenotypes in reverse-MR after multiple-testing correction^71–73^.

Concordance of MR estimates across instruments was assessed using Pearson correlation coefficients calculated both across all proteins and restricted to those meeting the stage 2 selection criteria. Effect estimates were reported per s.d. higher in GI_LSI_, per category higher in GI_CigDay_ and as the effect per 1-unit higher genetically predicted log-odds of ever smoking for GI_SmkInit._ Continuous outcomes are reported as *β* coefficients and binary outcomes as odds ratios (ORs).

#### Negative control analysis in UKB never-smokers

As a final screen for potential pleiotropy, weighted genetic scores for each genetic instrument were tested for associations with candidate proteins in never-smoking participants from the UKB proteomics subsample (data field: 30900; *n* = 54,219) as a negative control. Proteins were quantified on the Olink Explore 3072 panel as normalized protein expression values on a log_2_ scale; sample selection, processing and quality-control procedures have been described previously^18^.

Weighted genetic scores for the three smoking phenotypes were constructed using published GWAS effect estimates and tested for associations with candidate protein levels among never-smoking participants (data field: 20116) of European ancestry. Proteins showing a significant association after multiple-testing correction, and therefore strong evidence that the effects of the instrument do not operate through smoking behavior, were excluded. Models were adjusted for age (data field: 21022), age^2^, sex (data field: 31), assessment center (data field: 54), genotyping array batch (data field: 22000) and the first 20 genetic principal components (data field: 22009). Participants from the nonrandom proteomic subsample (batches 0 or 7; resource: 1016) and those with missing protein data were excluded, leaving up to 19,291 participants. Plasma protein levels were residualized for plate effects (resource: 1018) and rank-based inverse-normal-transformed using RNOmni (v.1.0.1.2).

#### Functional enrichment and tissue annotation

We characterized smoking-related proteins jointly and stratified them according to the genetically predicted smoking phenotype through which they were identified. GO and KEGG pathway enrichment analyses were conducted using clusterProfiler (v.4.2.2), applying hypergeometric tests (using FDR-corrected *P* < 0.05) to identify overrepresented biological processes and pathways^74–77^.

The Human Protein Atlas was used to identify proteins actively secreted into the plasma. Organ enrichment was assessed using GTEx (v.8) and defined as at least fourfold higher expression in one organ relative to others^78–80^.

### Stage 2: causal effects of smoking-related proteins on disease outcomes

#### Disease outcome data

To maximize statistical power in stage 2, disease outcome summary statistics were derived from fixed-effects IVW meta-analyses of the largest publicly available GWAS and the MVP, where the latter had not already been included. Meta-analysis was conducted in METAL using variant-level effect estimates and standard errors^81^. The resulting datasets included 322,987 cases and 1,441,499 controls for IHD; 242,283 cases and 1,569,734 controls for T2D; 161,613 cases and 1,252,808 controls for COPD; 83,293 cases and 1,654,140 controls for ischemic stroke; and 42,331 cases and 491,047 controls for lung cancer^82–88^.

#### *cis*-MR analyses

Genetic instruments for circulating plasma proteins were restricted to *cis*-pQTLs, defined as variants located within ±1 Mb of the gene encoding the protein and associated with protein levels at *P* < 1.7 × 10^−11^ (5 × 10^−8^/2,923). The same LD and MAF thresholds used for smoking instruments were applied. Associations were estimated using RE-IVW MR when at least two variants were available and the Wald ratio when only one variant was available.

#### Genetic colocalization

For smoking-related proteins with significant disease associations, Bayesian genetic colocalization was used to assess whether protein levels and disease shared a causal variant rather than being associated through LD. Colocalization was conducted within a ±250-kb window centered on the lead *cis*-pQTL, to capture predominantly *cis*-acting signals for each protein-coding gene, and excluded variants with an MAF < 0.01. ABF colocalization used default priors, assuming a prior probability of 1 × 10^−4^ that any variant was associated with either trait and 1 × 10^−5^ that a variant was associated with both traits^89^. Posterior probabilities were estimated for five hypotheses: (H_0_) no association with either trait; (H_1_ or H_2_) association with only one trait; (H_3_) association with both traits driven by distinct causal variants; or (H_4_) association with both traits driven by a shared causal variant.

Colocalization was also assessed using the SuSiE regression framework, which accommodates allelic heterogeneity by decomposing association signals into multiple independent single-effect components^90^. Evidence of a shared causal variant was assessed from overlap of credible sets between traits. Given differing assumptions regarding allelic heterogeneity, strong evidence (PP.H4 > 80%) from either ABF or SuSiE colocalization was sufficient for progression to stage 3.

### Stage 3: MVMR, replication and observational triangulation

#### MVMR

To assess whether a shared genetic architecture with vascular risk factors influenced associations between smoking and ASGR1 levels in stage 2, we first tested genetically predicted alcohol consumption, BMI, LDL levels, SBP, DBP and T2D against ASGR1 levels using univariable MR. Risk factors with significant associations (*P* < 0.05) were reevaluated using MVMR, modeled alongside the smoking variable. MVMR extends conventional MR by simultaneously modeling multiple exposures, enabling estimation of the direct effect of smoking while accounting for correlated risk factors^91^.

Genome-wide genetic instruments for vascular risk factors were obtained from the largest available consortia for each trait and constructed using the same selection criteria as the smoking instruments^66,85,92–94^. Conditional F-statistics were calculated to assess instrument strength in multivariable models.

#### Replication in independent proteomic cohorts

Smoking–ASGR1 (univariable and significant vascular risk factor MVMR) and ASGR1–IHD associations were evaluated for replication using independent proteomic datasets from ARIC (*n* = 7,213), deCODE (*n* = 35,559) and Fenland (*n* = 10,804), each profiled using the SomaScan v.4 platform^51,95,96^. Smoking–ASGR1 estimates from the UKB-PPP, deCODE and Fenland were meta-analyzed using the fixed-effect IVW method.

#### Tissue-specific QTL integration

The lead *cis*-pQTL for ASGR1, rs55714927, was queried in GTEx to identify regulatory variants within liver tissue^79^. Significant (*P* < 5 × 10^−8^) expression QTLs and sQTLs within the relevant enriched tissue were extracted; ABF and SuSiE colocalizations were performed between tissue-specific QTL signals, circulating ASGR1 levels and IHD.

#### Observational analyses in the UKB

We assessed observational associations between smoking and ASGR1 levels, and ASGR1 and IHD incidence, in the UKB proteomics subsample. The baseline assessments in the UKB collected detailed information on smoking, including frequency, age initiated and amount of tobacco smoked, along with a range of physical measurements^97^.

Associations between smoking status, smoking behaviors and ASGR1 levels were examined using multivariable linear regression, with estimated marginal means derived using the emmeans (v.2.0.0) R package. Smoking status was classified as never-smoker, former-smoker or current-regular smoker. Occasional smokers (data fields: 1239 and 1249) were classified as current-regular smokers if they had smoked more than 100 cigarettes in their lifetime (data field: 2644), and as never-regular smokers otherwise. Smoking behavior measures included the following categorized variables: cigarettes per day (none, <10, 10–19, 20–29, 30+, derived from data fields 2887, 3456 and 6183), duration of smoking (never-regular, <20, 20 to <30, 30 to <40, 40+ years, derived from data field 6194), age at smoking initiation (never-regular, 20+, 16–20, <16 years, derived from data fields 2867 and 3436) and pack-years (never-regular, <20, 20 to <30, 30 to < 40, 40+, defined as (cigarettes per day / 20) × smoking duration). We excluded individuals who were not part of the random subsample and those with missing data or responses of ‘do not know’ or ‘prefer not to answer’ for ASGR1 levels, smoking variables or covariates, leaving 37,070 participants for the cross-sectional analysis. ASGR1 levels were residualized for plate effects and rank-based inverse-normal-transformed. Models were adjusted for age, age^2^, sex, Townsend deprivation index (data field: 22189), assessment center, ethnicity (data field: 21000), alcohol consumption (data field: 20117), BMI (data field: 21001) and fasting time (data field: 74). Linear trends across ordered smoking categories were assessed using chi-squared tests for trend.

Associations between ASGR1 levels (analyzed continuously and as four equal groups) and incident IHD were evaluated using time-dependent Cox proportional hazards models, stratified according to age at risk (5-year bands between 40 and 85) and adjusted for smoking status and the aforementioned covariates. Incident IHD was defined using hospital admission or mortality records (International Statistical Classification of Diseases and Related Health Problems, Tenth Revision codes I20–I25; OPCS Classification of Interventions and Procedures version 4 procedure codes K234, K40–46, K471, K49–50 or K75). Follow-up time was calculated from the date of recruitment (data field: 53) to the date of first event, censoring or 15 years after recruitment, whichever occurred first. Participants with prevalent IHD at baseline were excluded (developed IHD before recruitment, doctor-diagnosed heart attack or angina (data field: 6150), an entry for age of heart attack diagnosed (data field 3894) or self-reported heart attack or angina (data field: 20002) during participant interview). After exclusions for batch, missing protein data or missing covariate data, 40,372 participants were included in the incident disease analyses. Cox models were fitted using the survival (v.3.7.0) R package. Kaplan–Meier curves stratified according to ASGR1 fourths were compared using the log-rank test.

### Stage 4: phenome-wide association and mediation analyses

#### Phenome-wide association study

We conducted a PheWAS to characterize the broader trait architecture of ASGR1. Initially, the lead *cis*-pQTL for ASGR1, rs55714927, was queried in the CMDKP, selected because of its broad coverage of well-powered cardiometabolic traits and diseases that may contribute to or arise as a consequence of cardiometabolic pathology. A total of 709 phenotypes derived from European-ancestry populations were evaluated. Associations between the *cis*-pQTL and traits or diseases were considered ASGR1-related if they passed Bonferroni correction (*P* < 7.05 × 10^−5^). Phenotypes that were sex-specific, adjusted for another phenotype or stratified according to disease status were excluded from further downstream analyses.

To investigate potential repurposing opportunities or safety events beyond cardiometabolic phenotypes captured in the CMDKP, the lead variant was additionally queried in the MVP, including 1,779 phenotypes^82^. Non-cardiometabolic outcomes passing Bonferroni correction (*P* < 2.81 × 10^−5^) were retained for the downstream analyses.

#### Two-step MR mediation analyses

Two-step MR was used to estimate the proportion of smoking–phenotype associations mediated by ASGR1 (ref. ^98^). First, the smoking–ASGR1 association (*β*_1_) from stage 1 and the ASGR1–outcome association (*β*_2_) from stage 2 (or estimated using *cis*-MR where required) were obtained. Second, two-sample MR was used to estimate the total effect of smoking on the outcome (*β*_TE_). The proportion mediated was calculated as (*β*_1_ × *β*_2_) / *β*_TE_, with standard errors and 95% CIs derived using the delta method^98^.

#### ASGR1 network mediation

Finally, we established a protein–protein interaction network to evaluate whether additional circulating proteins mediated associations between ASGR1 and its downstream phenotypes. Protein–protein interaction networks were constructed by identifying *trans*-acting protein associations for the ASGR1 lead *cis*-pQTL using proteogenomic data from deCODE, to avoid sample overlap. Candidate network proteins were required to demonstrate shared genetic architecture using colocalization (PP.H4 > 0.80) and correct causal direction using Steiger filtering.

Network mediation was assessed using the same two-step MR framework, where *β*_1_ represented the association between ASGR1 and the network protein, *β*_2_ the association between the network protein and the downstream trait and *β*_TE_ represented the ASGR1–trait association. Network-mediated effects were excluded if *β*_2_ showed evidence of horizontal pleiotropy (MR-Egger intercept *P* < 0.05) or reverse causation. Mediation estimates were reported only when *β*_1_, *β*_2_ and *β*_TE_ were directionally consistent to ensure interpretability^99^.

#### Software

All analyses were performed using R (v.4.4.2). MR analyses, including data harmonization, LD clumping and causal effect estimation, were conducted using TwoSampleMR (v.0.6.24), MR-PRESSO (v.1.0) and MVMR (v.0.4). Bayesian genetic colocalization was performed using coloc (v.5.2.3) for ABF and susieR (v.0.12.35) for SuSiE. Data visualization and figure generation were performed using VennDiagram (v.1.7.3), ggplot2 (v.4.0.1), circlize (v.0.4.17), enrichplot (v.1.26.6), locuszoomr (v.0.3.8) and survminer (v.0.5.1).

### Reporting summary

Further information on research design is available in the Nature Portfolio Reporting Summary linked to this article.

## Supplementary information

Reporting Summary

Peer Review File

Supplementary Tables 1–28.

## Data Availability

We used publicly available GWAS summary statistics from the following sources: smoking initiation, cigarettes per day and drinks per week from GSCAN (https://conservancy.umn.edu/items/91f6a003-6af2-4809-9785-53dc579dc788); lifetime smoking index from the UKB (https://data.bris.ac.uk/data/dataset/10i96zb8gm0j81yz0q6ztei23d); plasma proteome GWAS from the UKB (https://www.synapse.org/Synapse:syn51364943/files/), deCODE (www.decode.com/summarydata/) and Fenland (https://www.synapse.org/Synapse:syn51761394/wiki/622766); IHD disease from CARDIoGRAMplusC4D (https://cardiogramplusc4d.org/) and MVP (GCST90475929 at www.ebi.ac.uk/gwas/studies/); type 2 diabetes from T2DGGI (www.diagram-consortium.org/); COPD from (GCST90399694 at www.ebi.ac.uk/gwas/studies/) and MVP (GCST90476027 at www.ebi.ac.uk/gwas/studies/); ischemic stroke from GIGASTROKE (GCST90104540 at www.ebi.ac.uk/gwas/studies/) and MVP (GCST90477988 at www.ebi.ac.uk/gwas/studies/); lung cancer from a GWAS meta-analysis (GCST004748 at www.ebi.ac.uk/gwas/studies/) and MVP (GCST90475573 at www.ebi.ac.uk/gwas/studies/); BMI from GIANT (https://giant-consortium.web.broadinstitute.org/GIANT_consortium); SBP and DBP from a GWAS meta-analysis (GCST90310294 at www.ebi.ac.uk/gwas/studies/); CMDKP-identified traits sourced from GWAS conducted in the UKB (albumin, apolipoprotein B, AST, gamma-glutamyl transferase, HbA1c, IGF-1, monocyte percentage, nucleated RBC count, nucleated RBC percentage, total protein): https://personal.broadinstitute.org/ryank/Jurgens_Pirruccello_2022_GWAS_Sumstats.zip); ALP: GCST90013406 at www.ebi.ac.uk/gwas/studies/); C-reactive protein: GCST90029070 at www.ebi.ac.uk/gwas/studies/; sex-hormone-binding globulin: GCST90012111 at www.ebi.ac.uk/gwas/studies/; the Global Lipids Genetics Consortium (www.lipidgenetics.org/; MVP (DBP: GCST90475255); hypercholesterolemia: GCST90475721; hyperlipidemia: GCST90475718; mean corpuscular hemoglobin: GCST90475446; myocardial infarction: GCST90475932; and red blood cell distribution width: GCST90476361 at www.ebi.ac.uk/gwas/studies/; monocyte count (GCST90002340 at www.ebi.ac.uk/gwas/studies/); and serum uromodulin levels (aptamer-based assay: https://nxc-1453.imbi.uni-freiburg.de/s/gReQNkMJtkYYLxa). Enrichment analyses were informed from the Gene Ontology Resource (www.geneontology.org/) and the Kyoto Encyclopedia of Genes and Genomes (www.genome.jp/kegg/). For the gene expression data, we used GTEx (https://gtexportal.org/home/). Secretion info was obtained from the Human Protein Atlas (www.proteinatlas.org/). Individual-level data of the UKB can be accessed by following theis data access guidelines available at www.ukbiobank.ac.uk/enable-your-research.

## Code availability

The custom code used for the primary MR analysis is available via GitHub (https://github.com/linc5345/Smoking_ASGR1_IHD/). For further details about the code used in the analyses, please contact the corresponding authors.
