# Supplementary material for: Proteome-wide genetic analyses identify ASGR1 as a potential biomarker linking smoking with ischemic heart disease
Source: Nat Cardiovasc Res. 2026 Sep 22;5(10):999–1015. doi: 10.1038/s44161-026-00871-x (PMC13658040; doi:10.1038/s44161-026-00871-x)
Supplement: Supplementary file 1 [file 44161_2026_871_MOESM1_ESM.pdf]

nature portfolio

Corresponding author(s): Fiona Bragg, Zhengming Chen

Last updated by author(s): Jun 1, 2026

Reporting Summary

Nature Portfolio wishes to improve the reproducibility of the work that we publish. This form provides structure for consistency and transparency in reporting. For further information on Nature Portfolio policies, see our Editorial Policies and the Editorial Policy Checklist.

Statistics

For all statistical analyses, confirm that the following items are present in the figure legend, table legend, main text, or Methods section.

n/a	Confirmed	
☐	☒	The exact sample size (*n*) for each experimental group/condition, given as a discrete number and unit of measurement
☐	☒	A statement on whether measurements were taken from distinct samples or whether the same sample was measured repeatedly
☐	☒	The statistical test(s) used AND whether they are one- or two-sided<br>*Only common tests should be described solely by name; describe more complex techniques in the Methods section.*
☐	☒	A description of all covariates tested
☐	☒	A description of any assumptions or corrections, such as tests of normality and adjustment for multiple comparisons
☐	☒	A full description of the statistical parameters including central tendency (e.g. means) or other basic estimates (e.g. regression coefficient) AND variation (e.g. standard deviation) or associated estimates of uncertainty (e.g. confidence intervals)
☐	☒	For null hypothesis testing, the test statistic (e.g. *F*, *t*, *r*) with confidence intervals, effect sizes, degrees of freedom and *P* value noted<br>*Give P values as exact values whenever suitable.*
☐	☒	For Bayesian analysis, information on the choice of priors and Markov chain Monte Carlo settings
☒	☐	For hierarchical and complex designs, identification of the appropriate level for tests and full reporting of outcomes
☐	☒	Estimates of effect sizes (e.g. Cohen's *d*, Pearson's *r*), indicating how they were calculated

Our web collection on statistics for biologists contains articles on many of the points above.

Software and code

Policy information about availability of computer code

Data collection	No software was used - data were already collected.
Data analysis	All statistical analyses were carried out using R (v4.2.2) and packages TwoSampleMR (v0.6.24), MRPRESSO (v1.0), MVMR (v0.4), clusterProfiler (v4.2.2), coloc (v5.2.3), susieR (v0.12.35), VennDiagram (v1.7.3), ggplot2 (v4.0.1), circlize (v.0.4.17), enrichplot (v1.26.6), locuszoomr (v0.3.8), emmeans (v2.0.0), survival (v3.7.0), RNOmni (v1.0.1.2), and survminer (v0.5.1). Code used in the analyses and a link to the perform_mr() function required for the primary analyses are available at: https://github.com/linc5345/Smoking_ASGR1_IHD.

For manuscripts utilizing custom algorithms or software that are central to the research but not yet described in published literature, software must be made available to editors and reviewers. We strongly encourage code deposition in a community repository (e.g. GitHub). See the Nature Portfolio guidelines for submitting code & software for further information.

Data

Policy information about availability of data

All manuscripts must include a data availability statement. This statement should provide the following information, where applicable:

- Accession codes, unique identifiers, or web links for publicly available datasets
- A description of any restrictions on data availability
- For clinical datasets or third party data, please ensure that the statement adheres to our policy

We used publicly available GWAS summary statistics from the following sources: smoking initiation, cigarettes per day, and drinks per week from GSCAN (https://

conservancy.umn.edu/items/91f6a003-6af2-4809-9785-53dc579dc788); the lifetime smoking index from UK Biobank (https://data.bris.ac.uk/data/dataset/10i96zb8gm0j81yz0q6ztei23d); plasma proteome GWAS from UK Biobank (http://ukb-ppp.gwas.eu/), deCODE (https://www.decode.com/summarydata/), and Fenland (http://www.omicscience.org/apps/pgwas); ischaemic heart disease from CARDIoGRAMplusC4D (https://cardiogramplusc4d.org/) and MVP (GCST90475929 at https://www.ebi.ac.uk/gwas/studies/); type 2 diabetes from T2DGGI (https://www.diagram-consortium.org/); COPD from (GCST90399694 at https://www.ebi.ac.uk/gwas/studies/) and MVP (GCST90476027 at https://www.ebi.ac.uk/gwas/studies/); ischaemic stroke from GIGASTROKE (GCST90104540 at https://www.ebi.ac.uk/gwas/studies/) and MVP (GCST90477988 at https://www.ebi.ac.uk/gwas/studies/); lung cancer from a GWAS meta-analysis (GCST004748 at https://www.ebi.ac.uk/gwas/studies/) and MVP (GCST90475573 at https://www.ebi.ac.uk/gwas/studies/); BMI from GIANT (https://giant-consortium.web.broadinstitute.org/GIANT_consortium); systolic and diastolic blood pressure from a GWAS meta-analysis (GCST90310294 at https://www.ebi.ac.uk/gwas/studies/); CMDKP-identified traits sourced from GWAS conducted in UK Biobank (albumin, apolipoprotein B, AST, GGT, HbA1c, IGF-1, monocyte percentage, nucleated RBC count, nucleated RBC percentage, total protein: https://personal.broadinstitute.org/ryank/Jurgens_Pirruccello_2022_GWAS_Sumstats.zip; ALP: GCST90013406 at https://www.ebi.ac.uk/gwas/studies/; C-reactive protein: GCST90029070 at https://www.ebi.ac.uk/gwas/studies/; and sex hormone binding globulin: GCST90012111 at https://www.ebi.ac.uk/gwas/studies/), the Global Lipids Genetics Consortium (https://www.lipidgenetics.org/), MVP (diastolic blood pressure: GCST90475255; hypercholesterolemia: GCST90475721; hyperlipidaemia: GCST90475718; mean corpuscular haemoglobin: GCST90475446; myocardial infarction: GCST90475932; and red blood cell distribution width: GCST90476361 at https://www.ebi.ac.uk/gwas/studies/), for monocyte count (GCST90002340 at https://www.ebi.ac.uk/gwas/studies/), and for serum uromodulin levels (aptamer-based assay) (https://nxc-1453.imbi.uni-freiburg.de/s/gReQNkMJtkYYLxa). Enrichment analyses were informed from the Gene Ontology Resource (https://www.geneontology.org/) and the Kyoto Encyclopedia of Genes and Genomes (https://www.genome.jp/kegg/). For gene expression data, we used GTEx (https://gtexportal.org/home/). Secretion info was obtained from the Human Protein Atlas (https://www.proteinatlas.org/). Individual-level data of the UK Biobank can be accessed by following the data access guidelines available at: (https://www.ukbiobank.ac.uk/enable-your-research).

Research involving human participants, their data, or biological material

Policy information about studies with human participants or human data. See also policy information about sex, gender (identity/presentation), and sexual orientation and race, ethnicity and racism.

Reporting on sex and gender	In all regression analyses of UK Biobank data, sex was used as a covariate.
Reporting on race, ethnicity, or other socially relevant groupings	In all regression analyses of UK Biobank data, self-reported ethnicity was used as a covariate. In genetic analyses, we analysed solely European-ancestry individuals to maximise statistical power and prevent confounding by population stratification.
Population characteristics	The UK Biobank is a large-scale population-based prospective study. Baseline recruitment took place between 2006-2010. Observational analyses involved cross-sectional analysis of 37,070 individuals with baseline variables including protein measures, and survival analyses of 40,372 participants.
Recruitment	N/A
Ethics oversight	All contributing studies received approval from their respective institutional review boards.

Note that full information on the approval of the study protocol must also be provided in the manuscript.

Field-specific reporting

Please select the one below that is the best fit for your research. If you are not sure, read the appropriate sections before making your selection.

☒ Life sciences ☐ Behavioural & social sciences ☐ Ecological, evolutionary & environmental sciences

For a reference copy of the document with all sections, see nature.com/documents/nr-reporting-summary-flat.pdf

Life sciences study design

All studies must disclose on these points even when the disclosure is negative.

Sample size	The sample sizes of the datasets used in the genetic analyses are provided in Supplementary Table 1. The sample sizes of the observational analyses are described in the methods section.
Data exclusions	Exclusions to genetic variants were based on predefined criteria such as removing non-GWAS significant (p > 5e-8) or non-independent (linkage disequilibrium, r > 0.001) variants. Proteins in Stage 2 of the analyses had to have a cis-pQTL present. In observational analyses, participants were excluded if they had missing exposure or covariate data or were a case for the outcome being investigated at baseline. Full details are provided in the methods section.
Replication	The discovery MR analysis to identify smoking-related proteins was replicated using independent cohorts deCODE and the Fenland study. When replicating protein-disease associations, the ARIC study was additionally used.
Randomization	N/A - this was not an interventional study.
Blinding	N/A - this was not an interventional study.

Reporting for specific materials, systems and methods

We require information from authors about some types of materials, experimental systems and methods used in many studies. Here, indicate whether each material, system or method listed is relevant to your study. If you are not sure if a list item applies to your research, read the appropriate section before selecting a response.

Materials & experimental systems

n/a	Involved in the study
☒	☐ Antibodies
☒	☐ Eukaryotic cell lines
☒	☐ Palaeontology and archaeology
☒	☐ Animals and other organisms
☒	☐ Clinical data
☒	☐ Dual use research of concern
☒	☐ Plants

Methods

n/a	Involved in the study
☒	☐ ChIP-seq
☒	☐ Flow cytometry
☒	☐ MRI-based neuroimaging

Plants

Seed stocks	N/A
Novel plant genotypes	N/A
Authentication	N/A
