# Supplementary material for: Proteome-wide genetic analyses identify ASGR1 as a potential biomarker linking smoking with ischemic heart disease
Source: Nat Cardiovasc Res. 2026 Sep 22;5(10):999–1015. doi: 10.1038/s44161-026-00871-x (PMC13658040; doi:10.1038/s44161-026-00871-x)
Supplement: Supplementary file 2 [file 44161_2026_871_MOESM2_ESM.pdf]

nature portfolio

Peer Review File

Proteome-wide genetic analyses identify ASGR1 as a potential mediator of smoking on ischaemic heart disease

Corresponding Author: Professor Zhengming Chen

This file contains all reviewer reports in order by version, followed by all author rebuttals in order by version.

Version 0:

Reviewer comments:

Reviewer #1

(Remarks to the Author)
This is an impressive paper which uses Mendelian randomisation and proteomics analysis to identify circulating proteins causally affected by smoking, smoking related proteins causally related to cardiometabolic diseases, and the proportion of the effect of smoking on these diseases mediated by the identified proteins.
Both genome-wide and cis-MR analyses were utilised at different stages of the analysis as deemed appropriate, multivariable MR was used to separate effects of BMI and alcohol consumption on outcomes from the effects of smoking, with two-step MR being used for mediation analyses.
Due consideration is given to the potential effects of horizontal pleiotropy when using genome wide methods and to confounding by LD in cis MR by incorporating colocalization analysis.
The findings in relation to the effects of smoking on ASGR1, and ASGR1 on ischaemic heart disease are internally consistent and convincing.
The authors incorporate a good discussion of the biology of ASGR1.
I have no suggested changes other than for the authors to go over the manuscript and see if they can be even more clear in distinguishing for the reader where genetic instruments for MR analysis were selected genome wide rather than in cis.
One point to note which was not discussed is that ASGR1, which is exclusively expressed in the liver, is exploited for targeted drug delivery of RNA based therapeutics through conjugation of these therapies to N-acetylgalactosamine (GalNAC) which is a high affinity ligand for ASGR1.

(Remarks on code availability)

Reviewer #2

(Remarks to the Author)
This is a well-designed multi-stage proteomic Mendelian randomization (MR) study aiming to detect molecular mediators of the effects of smoking on cardiometabolic disease. In Stage 1, the authors use proteome-wide MR to identify circulating proteins whose levels are associated with genetic predisposition to smoking. Proteins meeting pre-specified criteria are carried forward to Stage 2, where associations with cardiometabolic endpoints are tested using cis-MR. Stages 3 and 4 pursue replication and downstream follow-up analyses. The authors report ASGR1 as a promising mediator of smoking's effect on ischemic heart disease (IHD) and support this with several replication and network analyses. The proteome-wide discovery framework, prioritization of disease-relevant proteins using cis-MR and colocalization, and the attempts at replication and observational triangulation are clear strengths. The analytical approach is up-to-date and the manuscript is clearly written. My principal concern, detailed below, relates to the robustness of Stage 1 for establishing a causal effect of smoking on ASGR1.

Major Comments
1. The central claim of the paper is that ASGR1 mediates the effect of smoking on IHD. It should be noted, however, that the association between ASGR1 variants and IHD risk is already well-established in the literature, with the expected mechanism being through LDL-cholesterol lowering. This includes strong genetic evidence from a rare frameshift variant in an Icelandic population (Nioi et al., N Engl J Med 2016) with ASGR1 inhibitors being already in clinical development for

hypercholesterolemia and IHD (e.g. by Amgen). The ASGR1–IHD link is therefore not novel. The novelty of this paper rests mainly on Stage 1: the use of MR to establish that smoking causally increases ASGR1 levels. This makes Stage 1 both the most important and the most methodologically vulnerable part of the analysis.
Multi-locus MR instruments, such as those used here, which explain only 0.4-1.2% of variance in the smoking exposures, are notoriously susceptible to false-positive associations driven by horizontal pleiotropy. This risk is not eliminated by the standard sensitivity analyses employed (MR-Egger intercept, MR-PRESSO, weighted median/mode consistency).
A direct and feasible test of whether the smoking instruments act through smoking per se is available to the authors: if the instruments' effect on ASGR1 is genuinely mediated by smoking behavior, the association should be absent among never-smokers in UK Biobank. Never-smokers represent a natural negative control: any residual association of the smoking instruments with ASGR1 in this group would indicate pleiotropy rather than a smoking-mediated effect. The authors have access to individual-level UK Biobank data and should perform this analysis; not only for ASGR1 but as a general filtering step across the full Stage 1 pipeline.
2. The finding that the smoking–ASGR1 association strengthens after additionally adjusting for BMI in multivariable MR is difficult to interpret, and the current discussion does not adequately address this. The conditional F-statistics for the smoking instruments fall below 10 when BMI is included, which raises concerns for weak instrument bias that would be expected to shift the estimate to the null. If the authors assume this step to be due to a form of "negative confounding", they should elaborate and also present the effects of genetically proxied BMI on ASGR1.
3. Was there sample overlap between the exposure GWAS (GSCAN) and the outcome GWAS (UKB-PPP) in Stage 1? In two-sample MR, sample overlap biases effect estimates toward the observational association and inflates apparent instrument strength, which would be a particular concern here given the already modest variance explained by the instruments. This should be explicitly reported and the authors should pursue sensitivity analysis using an instrument from a non-UK Biobank source.
4. The failure to replicate the smoking–ASGR1 association in deCODE reinforces the concerns raised in Comment 1 about the reliability of Stage 1. The authors treat this as a secondary observation, but given that deCODE is by far the largest replication cohort, the absence of a significant result there should be prominently acknowledged and its implications for the main conclusions discussed.
5. Rather than restricting the PheWAS to the metabolic traits available in the CMDKP, I would recommend a formal phenome-wide analysis in UK Biobank, FinnGen, or the Million Veteran Program. This would provide broader phenotypic coverage including the detection of unexpected associations that might be relevant for safety or repurposing opportunities for ASGR1-targeting pharmacotherapies. Full PheWAS results (not only those passing Bonferroni correction) should be provided in a supplementary table.
In addition, the PheWAS results as currently reported in Supplementary Table 20 are ambiguous with regards to directionality. Do they represent the effects of higher or lower genetically proxied ASGR1 levels? Please clarify.
6. Beyond clinical endpoints, I would recommend an additional step testing the effects of ASGR1 on additional vascular risk factors (BMI, diabetes/hyperinsulinemia, dyslipidemia/LDL/ApoB, systolic and diastolic blood pressure, alcohol consumption). Furthermore, the effects of these risk factors on ASGR1 should also be tested in MR analyses. Very often, vascular risk factors are strongly interconnected, as are their molecular signatures. This would help establish ASGR1 as a specific smoking-mediated molecular driver of ischemic heart disease.
7. In the observational analyses, the IHD incidence appears low relative to other UK Biobank studies with comparable follow-up (e.g. Schuermans et al., Nat Cardiovasc Res 3, 1516–1530, 2024). The authors should verify their case definition and ascertainment strategy.
8. Please clarify whether fixed-effects or random-effects IVW was used in the MR analyses.
9. If feasible, formal integration of a tissue-relevant eQTL layer with MR could strengthen target attribution at the ASGR1 locus. The manuscript notes that rs55714927 is a cis-eQTL in eQTLGen, but given that ASGR1 is predominantly expressed in the liver and the authors discuss smoking-related transcriptional changes in liver-expressed genes, liver-specific eQTL data, such as those from Broadaway et al. (Am J Hum Genet 2024) or GTEx, would provide more informative triangulation.
10. Causal language should be toned down. e.g. describing the colocalization result as providing "robust evidence of causality" in the abstract is not warranted, especially given the discordant results between ABF and SuSiE colocalization methods.

Minor Comments
1. In Figure 5 and Supplementary Figure 2, the x-axis label "per SD increase in genetically proxied smoking instrument" does not apply to the smoking initiation estimates, which are on a binary ever/never scale. Please correct the axis label accordingly.
2. PP.H4 for the ASGR1-IHD colocalization is reported as 0.98 in Figure 1 but as 0.91 in the abstract and main text. Please reconcile.
3. Some additional numerical discrepancies. The Results report 19 protein–disease associations, but the disease-specific breakdown in the text sums to 18; and the associations listed in Supplementary Table 14 could not be fully reconciled with Figure 4.
4. The UKB-PPP Olink platform measures 2923 unique proteins, as reflected in the Bonferroni threshold stated in the Methods (5×10^{-8}/2923). However, the manuscript consistently refers to 2922 proteins throughout. Please clarify which figure is correct and ensure consistency.
5. ALP is expanded as "alanine phosphatase" in Figure 1. The correct term is alkaline phosphatase.
6. Ischaemic stroke is misspelled on page 7, line 177.
7. WMED in the Figure 2 legend is expanded as "weighted mediation"; the correct expansion is "weighted median."
8. In Figure 3c, "Male" and "Female" appear as tissue categories under organ enrichment. Please clarify.
9. The title of Supplementary Table 17 refers to LTA4H, but the table content pertains to ASGR1. Please correct.

Marios K. Georgakis

(Remarks on code availability)

Reviewer #3

(Remarks to the Author)
I co-reviewed this manuscript with the invited reviewer. The full report was submitted under the invited reviewer's account.

(Remarks on code availability)

Reviewer #4

(Remarks to the Author)
Comments
The study has great clinical merit. The authors used plasma proteomics for large UK biobank cohort and identified 108 significant targets associated with strong smoking status, predicting a number of chronic diseases, e.g. ischemic heart disease, T2DM, COPD, ischemic stroke and lung cancer. ASGR1 was the top target. The authors also provide a potential pathomechanism for ASGR1 by down-regulating more than 100 other proteins.
1) The major problem of the study is the conclusion that ASGR1 specifically promotes smoking-dependent ischemic heart disease. As shown by several studies (see below) ASGR1 is a general marker or trigger of CAD and IHD. So how can the authors be sure that ASGR1 upregulation is not only a consequence of the IHD initiated by smoking or by any other risk factor?
2) The following work should be cited and discussed in detail: CD63+-ASGR1+- extracellular vesicles and particles association with severity of MASLD and steatohepatitis and smoking further increased this association (PMID: 41521111).
3) Also the study on plasma proteomics to identify drug targets for ischemic heart disease, including ASGR1 as a target for therapeutic intervention (PMID: 37940228).
4) Your reference #74 (PMID: 27192541) shows that loss-of-function variant of ASGR1 lowers the risk of coronary artery disease – so it seems hardly be a marker or specific target of smoking but rather a marker of and target for CAD and IHD in general. This assumption is also supported by the reference mentioned in my comment #3 (PMID: 37940228).
5) From the literature it also seems that ASGR1 is related to inflammation (40 hits in PubMed), which is a very general trigger of all kind of CVD.

(Remarks on code availability)

Version 1:

Reviewer comments:

Reviewer #1

(Remarks to the Author)
The authors have addressed my comments and I have no further issues to raise.

(Remarks on code availability)

Reviewer #2

(Remarks to the Author)
I thank the authors for the effort to address my comments.

I am still confused with the approach used to address comment #1.

If I have navigated the tables correctly, the results of the negative control analysis among never smokers are presented in Supplementary Table 7:

Smoking variable Protein N Beta Standarderror P value Lower CI Upper CI
LSI ASGR1 19291 0.352646 0.098227 0.000331 0.160122 0.545171
SmkInit ASGR1 19291 0.193288 0.058605 0.000975 0.078423 0.308154

The effects of lifetime smoking index (LSI) on ASGR1 levels in the full cohort are in Supplementary Table 3:

Protein Uniprot_ID No. SNPs Beta Standarderror P value Lower CI Upper CI

ASGR1 P07306 126 0.540288 0.089129 1.35E-09 0.365594 0.714981

And the effects of smoking initiation (SmkInit) on ASGR1 levels in the full cohort are in Supplementary Table 4:

Protein Uniprot_ID No. SNPs Beta Standarderror P value Lower CI Upper CI
ASGR1 P07306 51 0.232135 0.054449 2.01E-05 0.125416 0.338854

The authors argue that LSI failed negative control filtering, but SmkInit survived as it did not reach significance after multiple testing correction in the negative control analysis.

If I do not oversee something, the beta of SmkInit among never smokers in 0.193 with a p-value of 0.000975, whereas the beta in the full cohort is 0.232 with a p-value of 2E-05. In other words, the association between the genetic instrument and ASGR1 levels is largely preserved in individuals who have never smoked, even if it does not meet the authors' multiple-testing threshold.

In my view, these results are difficult to reconcile with the interpretation that the effect is mediated primarily through smoking behavior and instead raise concerns that the observed association may reflect pathways independent of smoking (horizontal pleiotropy).

Consequently, this has implications for the interpretation of the results, as, in my view, the main conclusion that “Proteome-wide genetic analyses identify ASGR1 as a potential mediator of smoking on ischaemic heart disease” is not well-supported by the presented data.

(Remarks on code availability)

Reviewer #3

(Remarks to the Author)
The authors have satisfactorily addressed my previous concerns. I have no further comments and support acceptance.

(Remarks on code availability)
I reviewed the code repository. The repository contains a clear README file and provides scripts covering the main analytical framework.
I did not perform a full end-to-end rerun because the required external GWAS and proteomic data. Overall, the code is a useful resource. It may not include every additional analysis described in the revision, but I do not think this affects the overall assessment of the manuscript.

Reviewer #4

(Remarks to the Author)
Most of my comments have been addressed. The more balanced discussion represents already a significant improvement.
1) Although the authors have now added a few notions on overlap of pathomechanistic pathways between smoking and chronic disease that may affect ASGR1 expression levels and signaling, they should add a summary of their response to a separate limitations section highlighting these critical issues.

Your Response – We agree with the reviewer that ASGR1 is a pleiotropic cardiovascular mediator rather than a highly specific ‘smoking-dependent’ biomarker. Our MR results demonstrate that smoking operates upstream of this established pro-atherogenic pathway. Just as LDL cholesterol is a general driver of IHD that can be upregulated by poor diet, ASGR1 represents a pathway through which the detrimental effects of smoking translate into structural cardiovascular risk.
We also agree that the possibility of reverse causation or bidirectional relationships should be carefully considered. While we cannot completely exclude more complex pathways (e.g., smoking contributing to subclinical IHD processes that subsequently influence circulating ASGR1 levels), we found limited evidence supporting reverse causation. First, in expanded univariable and MVMR analyses of determinants of ASGR1 levels, several established smoking-related cardiovascular risk factors (including LDL cholesterol and blood pressure) were not independently associated with ASGR1 levels. Second, although not included in the manuscript, IHD itself was not associated with ASGR1 levels (n SNPs = 237; mean F-statistic = 80.6; β=0.01 [95% CI: -0.04, 0.05], p=0.71) in reverse-MR analyses.
Although absence of evidence does not exclude more complex pathways, these findings are more consistent with ASGR1 representing a downstream molecular consequence of smoking exposure that may contribute to IHD risk, rather than simply reflecting established IHD itself.

2) ok.
3) ok.
4) ok.
5) ok.

(Remarks on code availability)

Version 2:

Reviewer comments:

Reviewer #2

(Remarks to the Author)
I remain unconvinced by the interpretation of ASGR1 as a mediator of the effect of smoking on ischaemic heart disease. In my view, the findings are more consistent with horizontal pleiotropy. Both the genetic scores for smoking initiation and cigarettes per day remain associated with ASGR1 levels in never smokers, indicating that these associations cannot be driven by smoking behavior. The attenuation of the association after adjustment for BMI does not alter this interpretation. If anything, it suggests that BMI mediates part of the association between the smoking genetic score and ASGR1, further highlighting that the genetic score is not a specific instrument for smoking behavior.

That said, the authors have made a substantial effort to address all reviewers' comments through additional analyses and have adopted a more balanced interpretation of their findings. While I remain unconvinced by the proposed mechanistic interpretation, I appreciate the revisions and believe that the manuscript has improved substantially.

(Remarks on code availability)

Open Access This Peer Review File is licensed under a Creative Commons Attribution 4.0 International License, which permits use, sharing, adaptation, distribution and reproduction in any medium or format, as long as you give appropriate credit to the original author(s) and the source, provide a link to the Creative Commons license, and indicate if changes were made.
In cases where reviewers are anonymous, credit should be given to 'Anonymous Referee' and the source.
The images or other third party material in this Peer Review File are included in the article’s Creative Commons license, unless indicated otherwise in a credit line to the material. If material is not included in the article’s Creative Commons license and your intended use is not permitted by statutory regulation or exceeds the permitted use, you will need to obtain permission directly from the copyright holder.
To view a copy of this license, visit https://creativecommons.org/licenses/by/4.0/

29th May 2025

Nature Cardiovascular Research
Editorial & Production Office
One New York Plaza Suite 4500
New York, NY 10004-1562, USA

Dear Dr. Tavosanis,

Re: **Proteome-wide genetic analyses identify ASGR1 as a potential mediator of smoking on ischaemic heart disease (NCVR-2026-03-0382R1)**

Thank you for your email on the 23rd April regarding revisions to the above manuscript. We have now revised the manuscript in light of the reviewer's helpful comments and hope that we have addressed the issues raised satisfactorily and that the manuscript can now be considered for publication.

Yours sincerely,

Alexander Tinworth	Fiona Bragg	Zhengming Chen
DPhil Student	*Associate Professor*	*Professor of Epidemiology*

Response to reviewer’s comments

(NCVR-2026-03-0382)

We would like to thank the editorial board and the external reviewers for their additional review and helpful feedback which has helped improve the manuscript. We have incorporated the reviewer comments and updated our methodology and discussion to reflect these requests. Responses to the reviewer and editor comments are detailed below as non-bold text, along with the accompanying revisions to the paper. Line numbers indicating where changes have been made refer to the “tracked” changes version of the manuscript.

REVIEWER #1:

1. I have no suggested changes other than for the authors to go over the manuscript and see if they can be even more clear in distinguishing for the reader where genetic instruments for MR analysis were selected genome wide rather than in cis. One point to note which was not discussed is that ASGR1, which is exclusively expressed in the liver, is exploited for targeted drug delivery of RNA based therapeutics through conjugation of these therapies to N-acetylgalactosamine (GalNAC) which is a high affinity ligand for ASGR1.

Response – We thank the reviewer for their positive assessment of our work and helpful feedback. We have revised the manuscript to improve clarity regarding when genome-wide versus *cis*-acting genetic instruments across the different stages of the MR analyses (page 6, lines 133; page 8, lines 187-188; page 9, line 219). We have also incorporated discussion regarding the role of ASGR1 in RNA-based therapeutics (page 25, lines 621-622).

REVIEWERS #2 & 3:

1. The central claim of the paper is that ASGR1 mediates the effect of smoking on IHD. It should be noted, however, that the association between ASGR1 variants and IHD risk is already well-established in the literature, with the expected mechanism being through LDL-cholesterol lowering. This includes strong genetic evidence from a rare frameshift variant in an Icelandic population (Nioi et al., N Engl J Med 2016) with ASGR1 inhibitors being already in clinical development for hypercholesterolemia and IHD (e.g. by Amgen). The ASGR1–IHD link is therefore not novel. The novelty of this paper rests mainly on Stage 1: the use of MR to establish that smoking causally increases ASGR1 levels. This makes Stage 1 both the most important and the most methodologically vulnerable part of the analysis.
Multi-locus MR instruments, such as those used here, which explain only 0.4-1.2% of variance in the smoking exposures, are notoriously susceptible to false-positive associations driven by horizontal pleiotropy. This risk is not eliminated by the standard sensitivity analyses employed (MR-Egger intercept, MR-PRESSO, weighted median/mode consistency).
A direct and feasible test of whether the smoking instruments act through smoking per se is available to the authors: if the instruments’ effect on ASGR1 is genuinely mediated by smoking behaviour, the association should be absent among never-smokers in UK Biobank. Never-smokers represent a natural negative control: any

residual association of the smoking instruments with ASGR1 in this group would indicate pleiotropy rather than a smoking-mediated effect. The authors have access to individual-level UK Biobank data and should perform this analysis; not only for ASGR1 but as a general filtering step across the full Stage 1 pipeline.

Response – We thank the reviewers for this suggestion and agree that polygenic smoking instruments explaining a modest proportion of variance may be susceptible to horizontal pleiotropy, therefore warranting further investigation.

As suggested, we have now incorporated negative control analyses using never-smokers in UKB as an additional filtering step in Stage 1. This reduced the total number of smoking-related proteins passing Stage 1 from 108 to 93 (page 14, line 352-353), and all corresponding figures and supplementary materials have been updated accordingly (Figures 1-4, Supplementary Figure 1, Supplementary Tables: 3-5, 7-8). We additionally updated Stage 2 analyses to reflect the revised multiple testing burden (80/93 instead of 95/108 smoking-related proteins having an available *cis*-pQTL). Consequently, the number of significant protein-disease associations was reduced from 18 to 15 (page 15, line 377, Figure 4).

For ASGR1 specifically, the smoking initiation instrument remained robust and retained evidence of association in the revised analyses. However, the lifetime smoking index association did not pass the negative control filtering step and was therefore removed from the revised downstream analyses (reflected in Figure 5, Supplementary Figure 2, Table 1, Supplementary Tables 16-18, and 24).

2. The finding that the smoking–ASGR1 association strengthens after additionally adjusting for BMI in multivariable MR is difficult to interpret, and the current discussion does not adequately address this. The conditional F-statistics for the smoking instruments fall below 10 when BMI is included, which raises concerns for weak instrument bias that would be expected to shift the estimate to the null. If the authors assume this step to be due to a form of "negative confounding", they should elaborate and also present the effects of genetically proxied BMI on ASGR1.

Response – We thank the reviewers for this important point. The effect estimates for genetically-proxied BMI on ASGR1 have now been presented more clearly in Figure 5 and explicitly described in the Results section (page 16, lines 397-399).

We agree that the strengthening of the smoking-ASGR1 association after adjustment for BMI requires more detailed interpretation. In the univariable MR analyses, both genetically-proxied smoking initiation and BMI showed positive associations with ASGR1 levels. However, following mutual adjustment in the MVMR framework, the BMI association attenuated substantially to the null, whereas the smoking association strengthened. This pattern may be consistent with shared or correlated pathways between smoking and BMI that partially confound the direct effect of smoking in univariable analyses.

We acknowledge the reviewers' concern regarding instrument strength. The conditional F-statistic for smoking initiation decreased below 10 in the BMI-adjusted MVMR models (minimum conditional F-statistic 9.3). This likely reflects the substantial reduction in available smoking-associated variants between the univariable (n=248) and multivariable (n=66) analyses. As noted by the reviewers, weak instrument bias in MVMR would generally be expected to bias estimates towards the null, therefore the observed strengthening of the smoking estimate following BMI adjustment is unlikely to be explained solely by weak instrument bias. In contrast, the BMI instrument remained strong in the multivariable setting (conditional F-statistic 67.4), yet the BMI-ASGR1 association

attenuated to the null (in both primary analyses and replication in deCODE and Fenland). Similarly, in the smoking/BMI/T2D MVMR model (added as a result of Reviewer 2/3, Comment 6), the BMI association was only nominally significant in the discovery analysis and was not replicated whereas the smoking association upon BMI adjustment remained consistent across datasets. These findings have now been discussed in the revised manuscript (pages 22-23, lines 552-569) and presented in the updated display items (Figure 5, Supplementary Figure 2, Supplementary Tables 17-18).

3. Was there sample overlap between the exposure GWAS (GSCAN) and the outcome GWAS (UKB-PPP) in Stage 1? In two-sample MR, sample overlap biases effect estimates toward the observational association and inflates apparent instrument strength, which would be a particular concern here given the already modest variance explained by the instruments. This should be explicitly reported and the authors should pursue sensitivity analysis using an instrument from a non-UK Biobank source.

Response – We thank the reviewers for raising this important point. There was limited sample overlap between the exposure GWAS datasets and the UKB-PPP outcome GWAS used in Stage 1, specifically 1.3% for smoking initiation, 7.5% for the LSI, and 5.6% for cigarettes per day. These overlap percentages are now explicitly stated in the limitations section of the revised manuscript (page 26, lines 654-645). Although sample overlap in two-sample MR can bias estimates, the degree of overlap with the UKB-PPP cohort was relatively small, therefore we selected the primary smoking instruments including UKB and 23andMe participants to maximise statistical power in the discovery analyses.

To further address this concern, we present here the analyses of genetically-predicted smoking initiation and ASGR1 levels using the available summary statistics which did not include UKB or 23andMe participants where the association between genetically-predicted smoking initiation and ASGR1 levels remains significant (*n* SNPs = 46; mean F-statistic = 47.4; β=0.23 [95% CI: 0.07, 0.39], *p*=0.0054). The results were directionally consistent across weighted median (β=0.19 [95% CI: -0.03, 0.41], *p*=0.09), mode (β=0.26 [95% CI: -0.23, 0.75], *p*=0.31), and Egger estimates (β=0.07 [95% CI: -0.84, 0.98], *p*=0.88) with no evidence of directional pleiotropy (MR-Egger intercept, *p*=0.72). The effect size for the IVW estimate presented here is additionally very similar to the analyses using the full instrument (β=0.25 [95% CI: 0.15, 0.34], $p=2.26 \times 10^{-7}$), suggesting that this association is unlikely to be driven by sample overlap.

4. The failure to replicate the smoking–ASGR1 association in deCODE reinforces the concerns raised in Comment 1 about the reliability of Stage 1. The authors treat this as a secondary observation, but given that deCODE is by far the largest replication cohort, the absence of a significant result there should be prominently acknowledged and its implications for the main conclusions discussed.

Response – We acknowledge the issue raised but also note that the deCODE findings were directionally consistent with UKB-PPP and Fenland, and that in MVMR analyses incorporating BMI, the smoking-ASGR1 association strengthened across all three cohorts, including deCODE. As discussed in response to Reviewer 2/3 Comment 2, we interpret this pattern as being consistent with shared or correlated pathways between smoking and BMI that may attenuate the univariable smoking estimate. To further address the concerns raised, we have now included new meta-analyses of all three studies (page 17, lines 412-413; Supplementary Table 17), which showed a strong association between smoking and ASGR1 levels. The revised manuscript now more explicitly discusses the lack of

replication in deCODE in both the Discussion (page 22-23, line 554-557) and the limitations section (pages 25-26, lines 639-645).

5. Rather than restricting the PheWAS to the metabolic traits available in the CMDKP, I would recommend a formal phenome-wide analysis in UK Biobank, FinnGen, or the Million Veteran Program. This would provide broader phenotypic coverage including the detection of unexpected associations that might be relevant for safety or repurposing opportunities for ASGR1-targeting pharmacotherapies. Full PheWAS results (not only those passing Bonferroni correction) should be provided in a supplementary table.

In addition, the PheWAS results as currently reported in Supplementary Table 20 are ambiguous with regards to directionality. Do they represent the effects of higher or lower genetically proxied ASGR1 levels? Please clarify.

Response – Done. As suggested, we have now expanded the PheWAS of rs55714927:C:T using data from the MVP covering 1779 traits, including many beyond the metabolic traits available in the CMDKP. Full PheWAS results are provided in Supplementary Table 23 and mentioned in the Results (page 18, lines 452-454).

This additional PheWAS in the MVP did not identify additional associations suggestive of safety liabilities or repurposing opportunities beyond those observed in the CMDKP.

We have also clarified the directionality of the reported effects in both in Supplementary Table 22 (formerly 20) and Supplementary Table 23, with results representing the effects of genetically-proxied lower ASGR1 levels associated with the ASGR1-lowering (T) allele.

6. Beyond clinical endpoints, I would recommend an additional step testing the effects of ASGR1 on additional vascular risk factors (BMI, diabetes/hyperinsulinemia, dyslipidemia/LDL/ApoB, systolic and diastolic blood pressure, alcohol consumption). Furthermore, the effects of these risk factors on ASGR1 should also be tested in MR analyses. Very often, vascular risk factors are strongly interconnected, as are their molecular signatures. This would help establish ASGR1 as a specific smoking-mediated molecular driver of ischemic heart disease.

Response – For clarification, several of the requested vascular risk factors were already included within our analytical framework. Specifically, the effects of genetically-proxied ASGR1 on T2D was assessed in the Stage 2 analyses, whilst associations with BMI, LDL cholesterol, systolic and diastolic blood pressure, and alcohol consumption were evaluated within the PheWAS analyses.

To further address the reviewer's suggestion and better characterise the relationship between ASGR1 and vascular risk factors, we have expanded Stage 3 to include T2D, LDL, and blood pressure, in addition to BMI and alcohol consumption, in univariable MR analyses of vascular risk factors on ASGR1 levels. We additionally performed MVMR with smoking on risk factors demonstrating evidence of association with ASGR1 levels in univariable analyses ($p<0.05$; Figure 5, Supplementary Figure 2, Supplementary Table 18).

7. In the observational analyses, the IHD incidence appears low relative to other UK Biobank studies with comparable follow-up (e.g. Schuermans et al., Nat Cardiovasc Res 3, 1516–1530, 2024). The authors should verify their case definition and ascertainment strategy.

Response – As suggested, we have re-checked our IHD case definition and outcome ascertainment strategy and confirmed that the coding and event extraction were implemented as intended.

The incidence of IHD observed in in our study (3398 events among a cohort of 40,372 participants) is in fact slightly higher than the cited study (2729 events among a cohort of 44,313). One possible explanation for this difference relates to variation in disease definitions and included diagnostic codes. Our definition incorporated angina-related diagnoses (ICD-10 I20) whereas the cited study appears to have used a narrower CAD definition that excluded angina phenotypes. In addition, our definition included the OPCS-4 procedure codes K234 and K471 which were not included in the cited study, which may have also contributed to the discrepancy in event rates between studies.

8. Please clarify whether fixed-effects or random-effects IVW was used in the MR analyses.

Response – The IVW used in the study adopted the random-effects method to account for potential heterogeneity between instrumental variables. This has been clarified on page 6, line 142.

9. If feasible, formal integration of a tissue-relevant eQTL layer with MR could strengthen target attribution at the ASGR1 locus. The manuscript notes that rs55714927 is a cis-eQTL in eQTLGen, but given that ASGR1 is predominantly expressed in the liver and the authors discuss smoking-related transcriptional changes in liver-expressed genes, liver-specific eQTL data, such as those from Broadaway et al. (Am J Hum Genet 2024) or GTEx, would provide more informative triangulation.

Response – Done. As suggested, we have incorporated a tissue-specific QTL layer to Stage 3 of the analyses using data from GTEx.

Querying rs55714927 in the GTEx portal identified the variant as a liver-specific splicing QTL (sQTL) for ASGR1, with significant associations observed at two of the 5 annotated ASGR1 splice junctions. We subsequently performed colocalization analyses between these liver sQTL signals, circulating protein levels, and IHD risk. Both ABF and SuSiE colocalization analyses supported a shared causal variant between the two liver ASGR1 splicing signals and circulating ASGR1 levels, while ABF colocalization additionally supported a shared causal variant between these ASGR1 splicing signals and IHD risk. We have additionally revised the Discussion to incorporate these findings (page 21-22, lines 530-532 and page 22, lines 542-543).

10. Causal language should be toned down. e.g. describing the colocalization result as providing "robust evidence of causality" in the abstract is not warranted, especially given the discordant results between ABF and SuSiE colocalization methods.

Response – Done. We have now toned down the causal language in the Abstract (page 2, lines 40-41) and in the Discussion (page 20, line 493; page 21, line 521).

11. In Figure 5 and Supplementary Figure 2, the x-axis label "per SD increase in genetically proxied smoking instrument" does not apply to the smoking initiation

estimates, which are on a binary ever/never scale. Please correct the axis label accordingly.

Response – Done. Throughout the manuscript, figures, and tables, the definition has been corrected to reflect that effect estimates for genetically-proxied smoking initiation are now described as representing the effect per 1-unit higher genetically predicted log-odds of ever smoking.

12. PP.H4 for the ASGR1-IHD colocalization is reported as 0.98 in Figure 1 but as 0.91 in the abstract and main text. Please reconcile.

Response – Done. Figure 1 has been updated.

13. Some additional numerical discrepancies. The Results report 19 protein–disease associations, but the disease-specific breakdown in the text sums to 18; and the associations listed in Supplementary Table 14 could not be fully reconciled with Figure 4.

Response – Done. After including negative control filtering, there were 15 protein-disease associations. We have carefully reviewed and updated the Results text, Figure 4, and Supplementary Table 14 to ensure the numbers are now consistently reported throughout the manuscript.

14. The UKB-PPP Olink platform measures 2923 unique proteins, as reflected in the Bonferroni threshold stated in the Methods ($5 \times 10^{-8}/2923$). However, the manuscript consistently refers to 2922 proteins throughout. Please clarify which figure is correct and ensure consistency.

Response – The 2923 figure includes GLIPR1 which failed quality control in the original UKB-PPP study.[1] This has been clarified on page 5, lines 129-130.

15. ALP is expanded as "alanine phosphatase" in Figure 1. The correct term is alkaline phosphatase.

Response – Thanks for pointing this out, which has been corrected.

16. Ischaemic stroke is misspelled on page 7, line 177.

Response – This has been corrected.

17. WMED in the Figure 2 legend is expanded as "weighted mediation"; the correct expansion is "weighted median."

Response – This has been corrected.

18. In Figure 3c, "Male" and "Female" appear as tissue categories under organ enrichment. Please clarify.

Response – Whilst there are no longer any "Female" enriched tissue categories included. "Male" has been clarified as "Prostate".

19. The title of Supplementary Table 17 refers to LTA4H, but the table content pertains to ASGR1. Please correct.

Response – This has been corrected.

REVIEWER #4:

1. The major problem of the study is the conclusion that ASGR1 specifically promotes smoking-dependent ischemic heart disease. As shown by several studies (see below) ASGR1 is a general marker or trigger of CAD and IHD. So how can the authors be sure that ASGR1 upregulation is not only a consequence of the IHD initiated by smoking or by any other risk factor?

Response – We agree with the reviewer that ASGR1 is a pleiotropic cardiovascular mediator rather than a highly specific 'smoking-dependent' biomarker. Our MR results demonstrate that smoking operates upstream of this established pro-atherogenic pathway. Just as LDL cholesterol is a general driver of IHD that can be upregulated by poor diet, ASGR1 represents a pathway through which the detrimental effects of smoking translate into structural cardiovascular risk.

We also agree that the possibility of reverse causation or bidirectional relationships should be carefully considered. While we cannot completely exclude more complex pathways (e.g., smoking contributing to subclinical IHD processes that subsequently influence circulating ASGR1 levels), we found limited evidence supporting reverse causation. First, in expanded univariable and MVMR analyses of determinants of ASGR1 levels, several established smoking-related cardiovascular risk factors (including LDL cholesterol and blood pressure) were not independently associated with ASGR1 levels. Second, although not included in the manuscript, IHD itself was not associated with ASGR1 levels (*n* SNPs = 237; mean F-statistic = 80.6; β=0.01 [95% CI: -0.04, 0.05], p=0.71) in reverse-MR analyses.

Although absence of evidence does not exclude more complex pathways, these findings are more consistent with ASGR1 representing a downstream molecular consequence of smoking exposure that may contribute to IHD risk, rather than simply reflecting established IHD itself.

2. The following work should be cited and discussed in detail: CD63+-ASGR1+-extracellular vesicles and particles association with severity of MASLD and steatohepatitis and smoking further increased this association (PMID: 41521111).

Response – We thank the reviewer for highlighting this relevant study, which has now been incorporated into the Discussion on the biological interpretation of smoking -> ASGR1 levels (page 21, lines 523-527).

3. Also the study on plasma proteomics to identify drug targets for ischemic heart disease, including ASGR1 as a target for therapeutic intervention (PMID: 37940228).

Response – Done. This has also been included in the Discussion to highlight the broader relevance of ASGR1 to cardiovascular disease biology and its potential role as a therapeutic target for IHD (page 25, line 617).

4. Your reference #74 (PMID: 27192541) shows that loss-of-function variant of ASGR1 lowers the risk of coronary artery disease – so it seems hardly be a marker or specific target of smoking but rather a marker of and target for CAD and IHD in general. This assumption is also supported by the reference mentioned in my comment #3 (PMID: 37940228).

Response – We agree that existing evidence supports ASGR1 as a broader cardiovascular disease-related target rather than a smoking-specific marker. We have revised the Discussion to better reflect this interpretation and further contextualise our findings within the wider literature on ASGR1 and CAD/IHD biology (page 23, lines 563-566).

5. From the literature it also seems that ASGR1 is related to inflammation (40 hits in PubMed), which is a very general trigger of all kind of CVD.

Response – We agree with the reviewer that ASGR1 may be linked to broader inflammatory pathways relevant to cardiovascular disease. As smoking is strongly associated with systemic inflammation, inflammatory processes may contribute to the observed smoking-ASGR1 relationship.

We also acknowledge that ASGR1 is likely influenced by multiple determinants beyond smoking. We have revised the Discussion accordingly (page 23, lines 563-566).

References

1. Sun BB, Chiou J, Traylor M, Benner C, Hsu YH, Richardson TG, et al. Plasma proteomic associations with genetics and health in the UK Biobank. Nature. 2023;622(7982):329-38.

OXFORD POPULATION HEALTH

UNIVERSITY OF OXFORD

27th July 2026

Nature Cardiovascular Research
Editorial & Production Office
One New York Plaza Suite 4500
New York, NY 10004-1562, USA

Dear Dr. Tavosanis,

Re: **Proteome-wide genetic analyses identify ASGR1 as a potential biomarker linking smoking with ischaemic heart disease (NCVR-2026-03-0382B)**

Thank you for your email on the 8 July regarding further revisions to the above manuscript. We have revised the manuscript to address the reviewers' comments and hope that the remaining issues have been sufficiently addressed.

Yours sincerely,

Alexander Tinworth	Fiona Bragg	Zhengming Chen
DPhil Student	*Associate Professor*	*Professor of Epidemiology*

Response to reviewer's comments

(NCVR-2026-03-0382B)

We would like to thank the editorial board and the external reviewers for their additional review and helpful feedback which has helped further improve the manuscript. We have incorporated the reviewer comments and updated our Discussion to reflect these requests. Responses to the reviewer and editor comments are detailed below as non-bold text, along with the accompanying revisions to the paper. Line numbers indicating where changes have been made refer to the "tracked" changes version of the manuscript.

<u>REVIEWER #2:</u>

1. I thank the authors for the effort to address my comments. I am still confused with the approach used to address comment #1. If I have navigated the tables correctly, the results of the negative control analysis among never smokers are presented in Supplementary Table 7:

Smoking variable Protein N Beta Standarderror P value Lower CI Upper CI
LSI ASGR1 19291 0.352646 0.098227 0.000331 0.160122 0.545171
SmkInit ASGR1 19291 0.193288 0.058605 0.000975 0.078423 0.308154

The effects of lifetime smoking index (LSI) on ASGR1 levels in the full cohort are in Supplementary Table 3:

Protein Uniprot_ID No. SNPs Beta Standarderror P value Lower CI Upper CI
ASGR1 P07306 126 0.540288 0.089129 1.35E-09 0.365594 0.714981

And the effects of smoking initiation (SmkInit) on ASGR1 levels in the full cohort are in Supplementary Table 4:

Protein Uniprot_ID No. SNPs Beta Standarderror P value Lower CI Upper CI
ASGR1 P07306 51 0.232135 0.054449 2.01E-05 0.125416 0.338854

The authors argue that LSI failed negative control filtering, but SmkInit survived as it did not reach significance after multiple testing correction in the negative control analysis. If I do not oversee something, the beta of SmkInit among never smokers in 0.193 with a p-value of 0.000975, whereas the beta in the full cohort is 0.232 with a p-value of 2E-05. In other words, the association between the genetic instrument and ASGR1 levels is largely preserved in individuals who have never smoked, even if it does not meet the authors' multiple-testing threshold. In my view, these results are difficult to reconcile with the interpretation that the effect is mediated primarily through smoking behavior and instead raise concerns that the observed association may reflect pathways independent of smoking (horizontal pleiotropy). Consequently, this has implications for the interpretation of the results, as, in my view, the main conclusion that "Proteome-wide genetic analyses identify ASGR1 as a potential mediator of smoking on ischaemic heart disease" is not well-supported by the presented data.

Response – We agree that the negative control analysis warrants a more careful interpretation, particularly given the modest attenuation of the SmkInit and ASGR1 association in never-regular smokers relative to the full cohort two-sample IVW estimate.

As the reviewer notes, the association between the genetic instrument for smoking initiation (SmkInit) and ASGR1 remains evident among never-regular smokers, although it does not survive correction for multiple testing. We acknowledge that this pattern is not fully consistent with a purely smoking-mediated mechanism and may indicate some degree of horizontal pleiotropy.

However, we would note that the comparison between the full-cohort two-sample MR IVW estimate and the negative control estimate is not direct, given differences in covariate adjustment, sample selection, analytical framework, and the potential influence of residual linkage disequilibrium. Using the same analytical approach as the negative control analysis, the linear regression association of the SmkInit genetic instrument with ASGR1 among all participants was 0.30 (95% CI: 0.22, 0.38, $p = 5.77\times10^{-13}$), which suggests a greater degree of attenuation in the negative control setting (0.19 [0.08, 0.31, p = 0.00098]) than may be apparent from comparisons with the two-sample IVW estimate.

Despite this, we agree there remains evidence of pleiotropy, and our results suggest that BMI-related pathways may be an important contributor. When BMI is included as a covariate in the regression model, the estimate in never-regular smokers is attenuated from 0.19 (0.08, 0.31, p = 0.00098) to the null, 0.05 (95% CI: -0.05, 0.15, p = 0.35), whilst the association remains significant when including ever-regular smokers: 0.16 (95% CI: 0.08, 0.24, $p = 2.04\times10^{-5}$). A summary can be viewed in **Response to reviewers Table 1**. We interpret these findings as consistent with a mixture of vertical (given that smoking is associated with adiposity) and horizontal pleiotropy, in line with the possibility that BMI and smoking behaviours have overlapping genetic architecture and vascular risk pathways as noted in the previous review round by Reviewers 2 & 3 (comment 6).

These observations collectively emphasise the importance of the multivariable MR analyses. This is shown in **Figure 5** of the manuscript, which displays the robustness of the association between SmkInit and ASGR1 after conditioning on BMI. This pattern was also observed in the deCODE and Fenland analyses, both of which using SomaScan-derived measurements of ASGR1 (**Supplementary Figure 2**).

In response to the reviewer's concern, we have revised the Discussion (Page 21-22, lines 514-535) to discuss the negative control results and the potential pleiotropic role of BMI, where we now state:

"Although the association of $GI_{SmkInit}$ with ASGR1 passed negative control filtering, there remained some evidence of horizontal pleiotropy. Of the studied vascular risk factors, BMI and T2D were associated with higher ASGR1 levels, consistent with previous work implicating ASGR1 as a 'BMI-driven' protein.[19] BMI-related pathways may therefore have explained this residual association in never-regular smokers, given that smoking and BMI are correlated traits with partially overlapping genetic architecture. Indeed, adjustment for BMI in MVMR analyses strengthened the smoking-ASGR1 association while attenuating the BMI-ASGR1 association towards the null. Although the BMI association with ASGR1 levels in UKB remained nominally significant following mutual adjustment for BMI and T2D, this was not replicated in deCODE or Fenland. Similarly, while the univariable smoking-ASGR1 association did not reach statistical significance in deCODE, effect estimates were directionally consistent across cohorts, significant in meta-analysis with UKB-PPP and

Fenland, and strengthened following BMI adjustment, mirroring findings in UKB and Fenland. Together, these findings suggest that genetic instruments for smoking and BMI may capture overlapping or correlated pathways that partially obscure smoking-ASGR1 associations in univariable analyses. Although ASGR1 is likely influenced by determinants beyond smoking alone (e.g., T2D), our findings support smoking exposure as a potential contributor to ASGR1 regulation and suggest that ASGR1 may represent one pathway linking smoking to IHD risk. More broadly, these findings highlight the importance of accounting for correlated vascular risk factors in proteome-wide MR studies and demonstrate how multivariable modelling can refine interpretation of smoking-associated proteomic signatures."

In addition, as suggested, we have now moderated the language used in the title and in various other places throughout the manuscript (Page 2, line 43; Page 4, line 86; Page 25, lines 631-632) to better reflect the strength of the evidence provided by the presented research.

Response to reviewers Table 1. Associations of genetically-predicted smoking initiation and ASGR1 in two-sample MR and linear regression analyses in UKB.

Method	Full cohort	Never smokers
Two-sample MR discovery analyses	0.25 (0.15, 0.34), p=4.26x10^{-7}	NA
Linear regression*	0.30 (0.22, 0.38), p=5.77x10^{-13}	0.19 (0.08, 0.31), p=0.00098
Linear regression* + BMI adjustment	0.16 (0.08, 0.24), p=2.04x10^{-5}	0.05 (-0.05, 0.15), p=0.35

*adjusted for age, age^2, sex, assessment centre, 20 genetic principal components, and genotyping array.

REVIEWER 4:

1. Most of my comments have been addressed. The more balanced discussion represents already a significant improvement.
Although the authors have now added a few notions on overlap of pathomechanistic pathways between smoking and chronic disease that may affect ASGR1 expression levels and signaling, they should add a summary of their response to a separate limitations section highlighting these critical issues.

Response – Done (Page 24/25, lines 600-606). We now additionally state:

"ASGR1 should not be interpreted as a smoking-specific biomarker, but rather a pleiotropic cardiovascular mediator that may lie downstream of smoking and linked with multiple biological pathways. Although there was little evidence of a bidirectional effect of ASGR1 levels on IHD, we cannot exclude more complex pathways. Accordingly, our findings should be interpreted as evidence that smoking influences IHD risk in part through ASGR1-related atherogenic pathways, rather than proof of a uniquely smoking-dependent mechanism".

14 August 2026

Nature Cardiovascular Research
Editorial & Production Office
One New York Plaza Suite 4500
New York, NY 10004-1562, USA

Dear Dr Tavosanis,

Re: **Proteome-wide genetic analyses identify ASGR1 as a potential biomarker linking smoking with ischaemic heart disease (NCVR-2026-03-0382B)**

Thank you for your email on the 7 August regarding provisional acceptance of the above manuscript. We have responded to the reviewers' final comment and hope that the remaining issue has been sufficiently addressed.

Yours sincerely,

Alexander Tinworth
DPhil Student

Fiona Bragg
Associate Professor

Zhengming Chen
Professor of Epidemiology

Response to reviewer's comments

(NCVR-2026-03-0382B)

We would like to thank the editorial board and the external reviewer for their additional review and helpful feedback which has helped further improve the scientific rigour of the manuscript. Our response to the reviewer comment is detailed below as non-bold text.

<u>REVIEWER #2:</u>

1. I remain unconvinced by the interpretation of ASGR1 as a mediator of the effect of smoking on ischaemic heart disease. In my view, the findings are more consistent with horizontal pleiotropy. Both the genetic scores for smoking initiation and cigarettes per day remain associated with ASGR1 levels in never smokers, indicating that these associations cannot be driven by smoking behavior. The attenuation of the association after adjustment for BMI does not alter this interpretation. If anything, it suggests that BMI mediates part of the association between the smoking genetic score and ASGR1, further highlighting that the genetic score is not a specific instrument for smoking behavior.

That said, the authors have made a substantial effort to address all reviewers' comments through additional analyses and have adopted a more balanced interpretation of their findings. While I remain unconvinced by the proposed mechanistic interpretation, I appreciate the revisions and believe that the manuscript has improved substantially.

<u>Response</u> – We thank the reviewer for acknowledging the additional analyses and revisions undertaken to address this point. We agree that the negative control findings suggest that the smoking genetic instruments are not entirely specific to smoking behaviour and that some horizontal pleiotropy is present, and the importance of this is reflected in our explicit and detailed consideration of this point in the manuscript's Discussion. At the same time, as also discussed in the manuscript, the evidence presented supports the conclusion that ASGR1 plays an important role in linking smoking to a higher risk of IHD, including persistence of the smoking-ASGR1 association only in smokers after accounting for the shared genetic effects with BMI and observed strengthening (in several independent datasets) of the association between $GI_{SmkInit}$ and ASGR1 levels after accounting for BMI. Reflecting Reviewer 2's comment, we have retained cautious language throughout the revised manuscript (for example, referring to ASGR1 as a potential *link* between smoking and IHD) and now additionally incorporating the negative control analyses in the Abstract.
